# *miR-106b* regulates the proliferation and differentiation of neural stem/progenitor cells through Tp53inp1-Tp53-Cdkn1a axis

**DOI:** 10.1186/s13287-019-1387-6

**Published:** 2019-09-23

**Authors:** Xiaohuan Xia, Hongfang Lu, Chunhong Li, Yunlong Huang, Yi Wang, Xiaoyu Yang, Jialin C. Zheng

**Affiliations:** 1 Center for Translational Neurodegeneration and Regenerative Therapy, Shanghai Tenth People’s Hospital affiliated to Tongji University School of Medicine, Shanghai, 200072 China; 2 Collaborative Innovation Center for Brain Science, Tongji University, Shanghai, 200092 China; 3 Departments of Pharmacology and Experimental Neuroscience, University of Nebraska Medical Center, Omaha, NE 68198-5930 USA; 4 Department of Anesthesiology, Tongji Hospital affiliated to Tongji University School of Medicine, Shanghai, 200065 China; 5 Department of Pathology and Microbiology, University of Nebraska Medical Center, Omaha, NE 68198-5930 USA

**Keywords:** *miR-106*, Neural stem/progenitor cells, Proliferation, Differentiation, *Tp53inp1*, *Cdkn1a*

## Abstract

**Background:**

Recent studies suggested that *miR-17*~*106* family was involved in the regulation of neural stem/progenitor cells (NPCs). However, distinct function of each family member was reported in regulating stem cells within and without the brain. Hence, to investigate the roles of individual miRNAs in *miR-17*~*106* family and mechanisms underlying their effects on neurogenesis is important to extend our understanding in the CNS development.

**Methods:**

Here, we examined the influence of *miR-106a*/*b* on the proliferation, differentiation, and survival of embryonic NPCs using specific mimics and inhibitor. The targets of *miR-106a*/*b* were identified from miRNA target prediction database and confirmed by luciferase assay. Specific siRNAs were utilized to erase the effects of *miR-106a*/*b* on the expression levels of target genes.

**Results:**

A positive correlation was observed between the temporal reduction of *miR-106a*/*b* expression levels and the decline of NPC pools in vivo and in vitro. The perturbation of *miR-106*’s function approaches revealed that *miR-106b*, but not *miR-106a*, facilitated the maintenance of NPCs and repressed the generation of both neuronal and glial cells, without preference to a particular lineage. No effect was observed for *miR-106a*/*b* in NPCs’ survival. The influence of *miR-106b* on NPCs’ proliferation and differentiation is likely achieved by directly inhibiting the expression of Tp53inp1 and Cdkn1a, key components of Tp53inp1-Tp53-Cdkn1a axis.

**Conclusion:**

Our study demonstrated a novel axis, *miR-106b*-Tp53inp1-Tp53-Cdkn1a, in regulating the proliferation and differentiation of NPCs.

**Electronic supplementary material:**

The online version of this article (10.1186/s13287-019-1387-6) contains supplementary material, which is available to authorized users.

## Background

In the development of the vertebrate central nervous system (CNS), multipotent neural stem/progenitor cells (NPCs) generate various types of neurons and glia in a spatially and temporally conserved pattern [1]. Emerging evidences from a variety of approaches show that the maintenance and differentiation of NPCs are regulated in response to the interaction of extracellular signals with the intrinsic properties of NPCs [2, 3]. Although remarkable progress has been made in the identification of cell-intrinsic and cell-extrinsic factors, how these factors fine-tuned gene expression remains largely unknown. Recently, miRNA-mediated gene silencing has been proved as an essential mechanism on the regulation of multiple cellular biological processes.

From the discovery of first microRNA (miRNA) in 1993, miRNAs have emerged as important players in the control of stem cell behavior [4, 5]. miRNAs are evolutionary conserved small non-coding RNAs (22~24 nucleotides), which bind to partially complementary target sites in the 3′ untranslated region (3′UTR) of transcripts and regulate the expression of these transcripts in a post-transcriptional manner [6]. As one of the broadly conserved miRNA polycistron, *miR-17~106* family, including *miR-17*~*92* cluster and its two paralogs, *miR-106b~25* cluster and *miR-106a~363* cluster, is widely associated with multiple cellular processes, including proliferation, differentiation, and apoptosis [7–9]. *miR-17~106* family is firstly identified as oncogenic factors in a variety of tumor types by suppressing the expression of anti-tumor genes, such as *Cdkn1*a (*p21*), *Pten*, *Timp2*, *Smad7*, and *Bim* [10–13]. During the CNS development, the functional analyses of *miR-17*~*106* family in the regulation of NPCs majorly focus on the *miR-17*~*92* cluster which plays an important role in maintaining self-renewal and regulating neurogliogenic decision of NPCs [14–17]. Whether or not *miR-106b*~*25* and *miR-106a*~*363* clusters are also involved in the regulation of NPCs during cortical development remains largely unclear. More importantly, Naka-Kaneda et al. observed distinct roles of each *miR-17*~*106* family member in regulating neurogliogenesis in a controlled culture condition, suggesting the necessity to investigate the function of individual miRNAs in this family.

Here, we have examined the effects of *miR-106b* and *miR-106a*, key members of *miR-106b*~*25* and *miR-106a*~*363* clusters, on the regulation of embryonic NPCs and identified its target genes. Our observations suggested that *miR-106b*, but not *miR-106a*, is essential for the maintenance of NPCs’ proliferation and the repression of NSC’s differentiation. Moreover, we showed that *Tp53inp1* and *Cdkn1a* are direct targets of *miR-106b*. Finally, the loss of function approach for either *Tp53inp1* or *Cdkn1a* partially compromised the influence of forced *miR-106b* downregulation on the proliferation and differentiation of NPCs. Hence, our results highlight *miR-106b* as a key regulator to control the proliferation-differentiation balance of NPCs via Tp53inp1-Tp53-Cdkn1a axis.

## Methods

### Animal maintenance and use

C57BL/6J mice were housed and maintained in the Comparative Medicine Facility of the Tongji University School of Medicine (Shanghai, China). All procedures were conducted in accordance with the protocols approved by the Institutional Animal Care and Use Committee at the Tongji University School of Medicine.

### NPCs’ isolation, enrichment, and differentiation

Cortex from embryonic day 14 (E14) mice were dissected and dissociated as previously described. To generate neurospheres, dissociated E14 cortical cells were cultured for 3–4 days in NSC proliferation medium, containing NeuroCult® NSC Basal Medium (Stem Cell Technologies), NeuroCult® NSC Proliferation Supplements (Stem Cell Technologies), 20 ng/mL bFGF (BioWalkersville), 20 ng/mL EGF (BioWalkersville), 2 μg/mL heparin (Sigma), N2 supplement, 2 mM l-glutamine, 100 U/ml penicillin, and 100 μg/ml streptomycin. Neurospheres were collected, dissociated, and resuspended into single cells for a second-round neurosphere formation in suspension culture. After 3 rounds of selection and enrichment, neurosphere dissociates were cultured in suspension culture for NPCs’ proliferation experiments. For the NPCs’ differentiation experiments, neurosphere dissociates were cultured on Matrigel-coated 6-well plates or coverslips with NPCs’ differentiation medium, containing DMEM/F12, N2 supplement, 2% Knockout serum, 2 mM l-glutamine, 100 U/ml penicillin, and 100 μg/ml streptomycin. Both NPCs’ proliferation and differentiation experiments were terminated on 3 days after plating.

### miRNA mimics/inhibitor, Tp53inp1/Cdkn1a siRNA, and transfection

The mimics control, *miR-106b* mimics, inhibitor control, anti-miR-*106b* inhibitor, scrambled siRNA control, Tp53inp1 siRNA, and Cdkn1a siRNA were purchased from GenePharma (GenePharma Co., Ltd., Shanghai). Transfection of miRNA mimics/inhibitor or siRNAs was performed using the Lipofectamine 2000 reagent (Invitrogen) according to the manufacturer’s instruction.

### miRNA antagomir and administration

The antagomir negative control and antagomir-106b were purchased from GenePharma (GenePharma Co., Ltd., Shanghai). Fifty microliters of 100 μM antagomirs (antagomir negative control or antagomir-106b) was administrated intraperitoneally.

### Quantitative polymerase chain reaction

The mRNA and miRNA were isolated from cell and tissue samples using miRCURY RNA isolation kit (Exiqon, Woburn, MA). cDNA was synthesized using miScript II RT kit (Qiagen, Valencia, CA). Transcripts were amplified using SYBR green PCR kit (Qiagen, Valencia, CA) with the ABI7500 (Applied Biosystems, Waltham, MA). Sequences of transcript-specific primers are given in Additional file 1: Table S1. All qPCR results measured each sample in triplicate, and no-template blanks were used for negative controls. Amplification curves and gene expressions were normalized to the house-keeping gene Gapdh (for mRNA) and U6 snRNA (for miRNA).

### Immunofluorescence analysis

Immunofluorescence analysis for specific proteins was carried out as previously described [18]. Samples were incubated in primary antibody solutions (specifications shown in Additional file 1: Table S2) overnight at 4 °C. After washing with 1× PBS, samples were incubated with secondary antibodies (Cy3 or FITC) for 2 h at room temperature (RT). Samples were mounted using VectaShield (Vector Laboratories, Burlingame, CA), and images were taken using a Zeiss AX10 fluorescence microscope accompanied with ZEN 2.3 (blue edition) software. For quantification of the percentage of specific cell types in each experiment, the numbers of cell type-specific antigen-positive cells were counted in 15 randomly selected fields in 3 coverslips (5 fields each).

### TUNEL assay

Terminal deoxynucleotidyltransferase dUTP nick end labeling (TUNEL) was done on cultured cells using the In Situ Cell Death Detection Kit, TMR red (Sigma). Experiments were handled following the manufacturer’s protocol. Briefly, cells cultured on cover slips were fixed in 4% paraformaldehyde (PFA) at RT for 15 min. After two times washing with PBS, the cover slips were treated with a mixture of Label Solution and Enzyme Solution (10:1) for 1 h at 37 °C. Label Solution treatment was used as negative control. DNase I recombinant (1000 U/ml)-treated cover slips (10 min incubation at RT) were used as positive control. The cover slips were then washed twice in PBS. The cover slips were mounted using VectaShield (Vector Laboratories, Burlingame, CA) and processed to microscopy using a Zeiss AX10 fluorescence microscope accompanied with ZEN 2.3 (blue edition) software.

### EdU incorporation assay

DNA synthesis was performed by a Click-iT® EdU Imaging Kits (Thermo Scientific, #C10340) according to the manufacturer’s instructions. For in vivo studies, EdU was administrated intraperitoneally 8 h prior to sacrificing animals. For in vitro studies, NPCs were seeded on 6-well-plates (5 × 10^5^ cells/well) and cultured in NSC proliferation medium for 3 days. EdU was added 6 h prior to fixation. Cryostat tissue sections and cultured cells were fixed with 4% PFA for 15 min at RT, followed by permeabilization step using 0.5% Triton X-100. After permeabilization, the cells were incubated with Click-iT® reaction cocktails for 30 min in dark room. Subsequently, the cells were mounted using VectaShield (Vector Laboratories, Burlingame, CA). Images were taken using Zeiss AX10 fluorescence microscope and AxioVision Rel. 4.8 software.

### Dual-Luciferase Reporter Assay

The Cdkn1a 3′UTR and Cdkn1a-mut 3′UTR were synthesized by Genewiz (Genewiz, Suzhou, China) and cloned into the PmeI and SacI site of the pmirGLO vector (Promega, Beijing, China), downstream of the firefly luciferase gene. For the luciferase assay, 3,000 293T cells were cultured in 96-well plates with DMEM, 10% FBS, 100 μg/ml streptomycin, and 100 U/ml penicillin. After the confluency reached ~ 70%, the cells were co-transfected with the *miR-106b* mimics and either the Cdkn1a 3′UTR or Cdkn1a-mut 3′UTR dual-luciferase reporter vector. Serum-free Opti-MEM was used to prepare the transfection solution, and Lipofectamine 2000 reagent (Invitrogen) was used to facilitate the transfection according to the manufacturer’s instruction. At 24 h post-transfection, Dual-Luciferase® Reporter Assay System (Promega Corporation, Beijing, China) was used to determine the luciferase activities on SpectraMax M5 microplate readers (Molecular Devices). The activity of Renilla luciferase was used to normalize that of firefly luciferase.

### Gene Ontology analysis

Mouse *miR-106b* predicted target genes for Gene Ontology (GO) analyses were extracted from Targetscan.org (http://www.targetscan.org/vert_72/). DAVID bioinformatics platform (david.ncifcrf.gov/home.jsp) and Panther Classification System (http://www.geneontology.org/) were used for GO analyses. *Mus musculus* genome data was used as annotation background. Biological_Process was selected as Functional_Database for gene function classification. Minimum and maximum numbers of genes in the category were set at 2 and 1000, respectively. Benjamini and Hochberg multiple test adjustment was used to adjust *P* value of analysis: *P* value < 0.05 was considered a significantly enriched pathway.

### Statistical analysis

Statistical analysis was performed using unpaired two-tail *t* test for pairwise comparisons (GraphPad Prism Software). Data were shown as mean ± SD, and *P* values < 0.05 were considered significant.

## Results

### *miR-106b* is highly expressed in NPCs

**Fig. 1:**
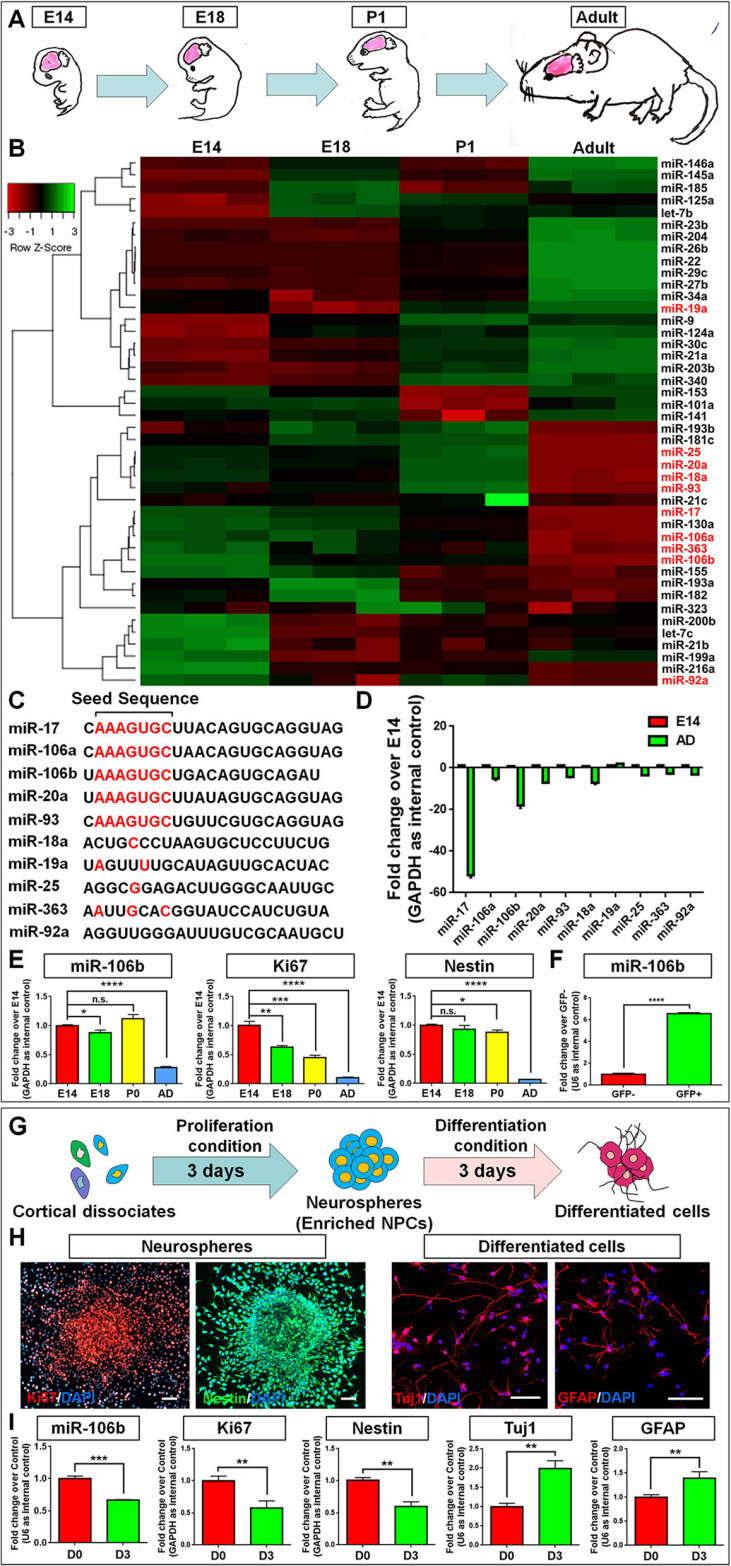
The temporal expression patterns of *miR-106b* correspond with the decline of proliferating NPC pool. **a** A schematic representation of the sample collection during brain development. **b** Hierarchical cluster analysis of 44 miRNAs expressed during cortical development. **c** Sequence comparison of miRNAs encoded by the *miR-17*~*106* family. **d** qPCR analysis of each miRNA in *miR-17*~*106* family in E14 and adult mouse cortexes. **e** The expression levels of the *miR-106b* decreased with time during brain development, positively correlated with that of transcripts corresponding to proliferating NPC markers, *Nestin* and *Ki67*. **f** qPCR analysis of expression levels of *miR-106b* in Nestin-GFP^+^ and Nestin-GFP^−^ cells. **g** A schematic representation of the enrichment and differentiation of NPCs. **h** The enrichment and differentiation of NPCs was confirmed by immunoreactivities corresponding to NPCs (Nestin/Ki67) and differentiated cells (Tuj1/GFAP), respectively. **i** The expression levels of *miR-106b* decreased during NPCs’ differentiation, positively correlated with that of transcripts corresponding to *Nestin* and *Ki67*. Scale bar, 20 μm (**g**). Data are mean ± SD. ∗∗∗∗*p* < 0.0001, ∗∗∗*p* < 0.001, ∗∗*p* < 0.01, and ∗*p* < 0.05

In order to identify candidate miRNAs that may be involved in the regulation of NPCs, we first determined the temporal expression patterns of 44 highly conserved miRNAs during cortical development by qPCR (Fig. 1a). Among these miRNAs, the expression levels of *miR-17*~*106* family members (labeled as red) showed significant reduction in adult stage, compared with developmental stages, except that of *miR-19a* (Fig. 1b). The sequence alignment suggested that *miR-17*, *miR-106a*, *miR-106b*, *miR-20a*, and *miR-93* share same seed sequence, while other miRNAs in this family were lack of similarity in sequence, implying those 5 miRNAs may be key factors for achieving the function of *miR-17*~*106* family in development (Fig. 1c). Expression analysis further identified two miRNAs (*miR-17* and *miR-106b*) in this family which showed more than 10-fold decrease in their levels during cortical development (Fig. 1d). Although the effects of *miR-17* and *miR-17*~*92* cluster in the regulation of NPCs and in CNS development were well investigated [15–17, 19], it remained largely unknown whether or not *miR-106b* exhibit similar function as *miR-17*. Our results suggested that the expression of *miR-106b* could be detected from early developing cortex (E14) (Fig. 1e). Afterwards, their expression decreased steadily, reaching their minimum levels in the adult stage. The temporal expression patterns of *miR-106b* were positively correlated with the decline of NPC pool during cortical development, confirmed by the expression transcripts corresponding to NPCs and proliferation markers *Nestin* and *Ki67*, respectively, implying the relationship of *miR-106b* expression with the regulation of NPCs (Fig. 1e). To confirm that *miR-106b* is also abundantly expressed in NPCs, we used embryonic Nestin:EGFP mice [20] and to sorted GFP^+^ and GFP^−^ cells from the brain (Fig. 1f). qPCR analysis demonstrated that the expression levels of *miR-106b* are significantly higher in GFP^+^ cells than GFP^−^ ones, suggesting the enrichment of *miR-106b* in Nestin^+^ NPCs.

We next determined the expression patterns of *miR-106b* in NPCs and differentiated cells in vitro (Fig. 1g). E14 cortical dissociates were cultured in the presence of EGF and FGF2, and neurospheres enriched in Ki67^+^/Nestin ^+^ cells were generated 3 days after plating, suggesting the enrichment of NPCs (Fig. 1h). NPCs, cultured in differentiation conditions for 3 days, differentiated into Tuj1^+^ neuronal and GFAP^+^ glial cells. qPCR results suggested that *miR-106b* were abundantly expressed in NPCs but with low expression in differentiated cells, sharing the same expression patterns of transcripts corresponding to *Nestin* and *Ki67* (Fig. 1i). Hence, the in vivo and in vitro studies demonstrated a corresponding positive correlation of *miR-106b* expression with the maintenance of NPCs, suggesting its functional involvement in the regulation of NPCs.

### *miR-106b* facilitates the proliferation and self-renewal of NPCs

**Fig. 2:**
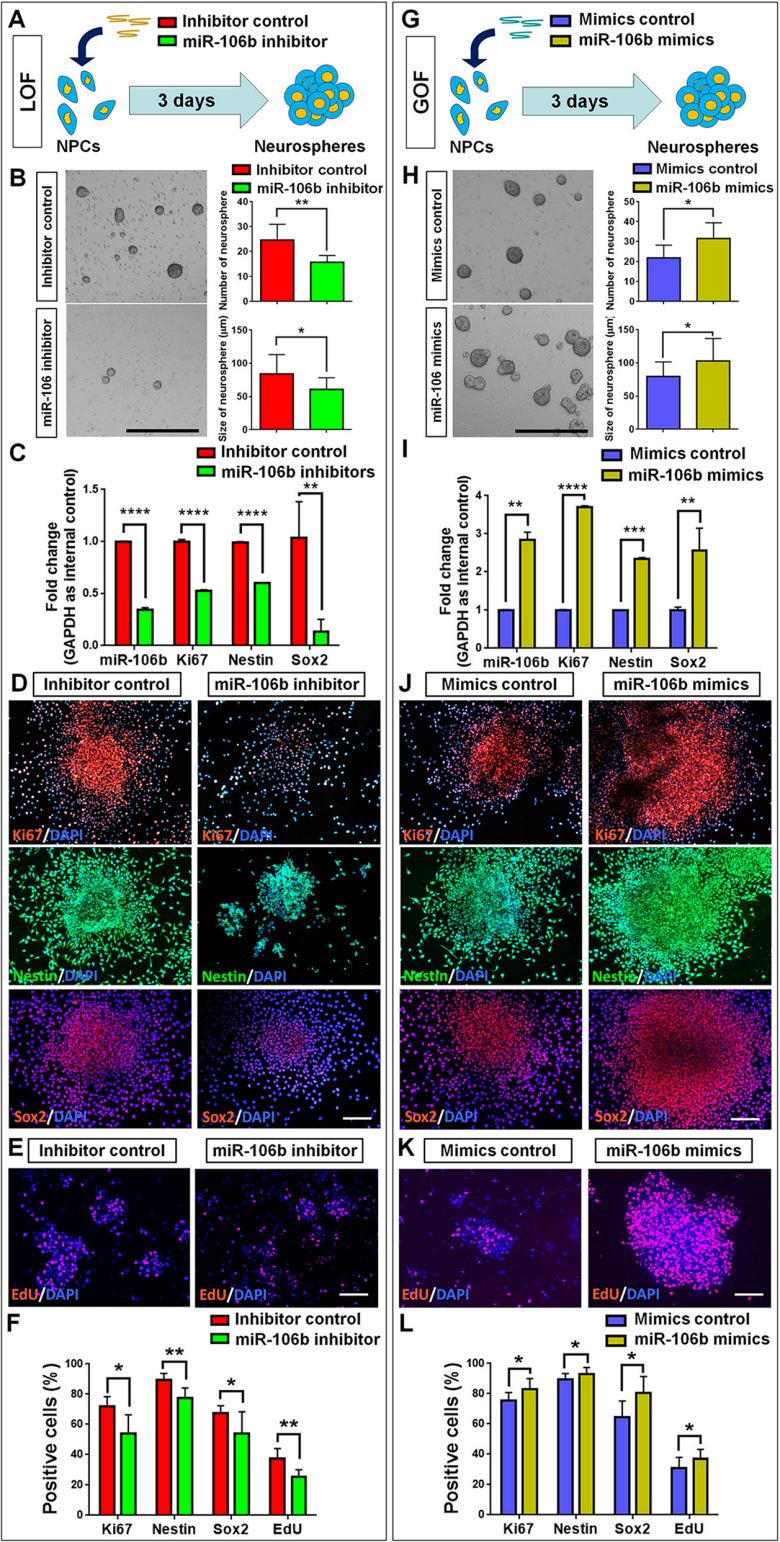
*miR-106b* facilitates the proliferation of NPCs. **a** A schematic representation of *miR-106b* LOF approach. **b** The number and size of neurospheres were quantified in the *miR-106b* LOF group, compared to controls. **c** qPCR analysis of expression levels of *miR-106b* and transcripts corresponding to markers of proliferating cells (*Ki67*) and NPCs (*Nestin*/*Sox2*) in the *miR-106b* LOF group, compared to controls. **d** Immunofluorescence analysis of transduced cells displaying proliferating cell (Ki67/EdU)- and NPC (Nestin/Sox2)-specific immunoreactivities. **e** Immunofluorescence analysis of transduced cells displaying EdU immunoreactivities. **f** Quantification of cells displaying immunoreactivities corresponding to proliferating cells and NPCs in the *miR-106b* LOF group, compared to controls. **g** A schematic representation of *miR-106b* GOF approach. **h** The number and size of neurospheres were quantified in the *miR-106b* GOF group, compared to controls. **i** qPCR analysis of expression levels of *miR-106b* and transcripts corresponding to markers of proliferating cells (*Ki67*) and NPCs (*Nestin*/*Sox2*) in the *miR-106b* GOF group, compared to controls. **j** Immunofluorescence analysis of transduced cells displaying proliferating cell (Ki67/EdU)- and NPC (Nestin/Sox2)-specific immunoreactivities. **k** Immunofluorescence analysis of transduced cells displaying EdU immunoreactivities. **l** Quantification of cells displaying immunoreactivities corresponding to proliferating cells and NPCs in the *miR-106b* GOF group, compared to controls. Scale bar, 400 μm (**b**, **h**) and 50 μm (**d**, **e**, **j**, **k**). Data are mean ± SD. ∗∗∗∗*p* < 0.0001, ∗∗∗*p* < 0.001, ∗∗*p* < 0.01, and ∗*p* < 0.05. Experiments were carried out three times in triplicates for in vitro perturbation

To understand the roles of *miR-106* in the regulation of NPCs, we firstly investigated the involvement of *miR-106b* in the proliferation of NPCs. Both *miR-106b* loss-of-function (LOF) and gain-of-function (GOF) approaches were carried out using *miR-106b*-specific inhibitor and mimics, respectively. In the *miR-106b* LOF approach, NPCs were transfected with either *miR-106b* inhibitor (=LOF group) or inhibitor control and cultured in proliferation conditions for 3 days in vitro (DIV) (Fig. 2a). The efficiency of transfection is validated by qRT-PCR, where significant downregulation of *miR-106* expression levels was observed in *miR-106b* LOF group, compared to controls (Fig. 2c). We observed a significant decrease in the number and size of neurospheres, demonstrating reduced proliferation and self-renewal of NPCs in the *miR-106b* LOF group, versus controls (Fig. 2b). Additionally, qRT-PCR analysis revealed a significant decline in the expression levels of transcripts corresponding to proliferation- and NPC-specific markers *Ki67* and *Nestin/Sox2*, respectively (Fig. 2c). A similar effect of *miR-106b* was observed on the proliferation capacity of NPCs. The proportions of cells expressing immunoreactivities corresponding to Ki67, Nestin, and Sox2 were significantly decreased in the *miR-106b* LOF group, compared to controls (Fig. 2d, f). The immunocytochemical analysis was corroborated with EdU (5-ethynyl-2-deoxyuridine) assay, that the proportion of EdU^+^ cells was significantly reduced in the *miR-106b* LOF group, versus controls, suggesting *miR-106b* LOF represses the proliferation of NPCs (Fig. 2e, f).

*miR-106b* GOF was carried out using same strategy of *miR-106b* LOF, where NPCs were transfected with either *miR-106b* mimics (=GOF group) or mimics control and cultured in proliferation conditions for 3 DIV (Fig. 2g). qRT-PCR analysis showed a significant increase in the levels of *miR-106b*, validating the transfection (Fig. 2i). In contrast to *miR-106b* LOF, ectopic expression of *miR-106b* accelerated the proliferation and self-renewal of NPCs, which led to more and bigger neurospheres; the upregulation of *Ki67*, *Nestin*, and *Sox2* transcript levels; and the elevation of proportions of cells with Ki67/Nestin/Sox2/EdU-specific immunoreactivities (Fig. 2h–l). Thus, our results indicated that *miR-106b* positively regulates the proliferation of NPCs.

In order to examine whether or not the effects of *miR-106b* were unique, we next examined the involvement of *miR-106b* paralog, *miR-106a*, in the proliferation of NPCs. The efficiency for *miR-106a* LOF and GOF was validated by qRT-PCR (Additional file 1: Figure S1C, I). Unlike *miR-106b*, the LOF and GOF of *miR-106a* did not significantly change the numbers and size of neurospheres, generated from NPCs (Additional file 1: Figure S1A, B, G, H). qRT-PCR analysis revealed an inverse correlation in the expression levels of *miR-106a* and transcripts corresponding to *Ki67*, *Nestin*, and *Sox2* (Additional file 1: Figure S1C, I). However, the immunofluorescence analysis suggested no significant difference in the proportions of cells with Ki67/Nestin/Sox2/EdU-specific immunoreactivities in both *miR-106a* LOF and GOF groups versus controls, although a significant decline was observed in the proportions of cells with Ki67- and Sox2-specific immunoreactivities in *miR-106a* LOF group (Additional file 1: Figure S1D-F, J-L). Thus, our observations suggested that *miR-106b*, but not *miR-106a*, may serve as a master regulator to maintain the stemness of NPCs.

### *miR-106b* inhibits the differentiation of NPCs

**Fig. 3:**
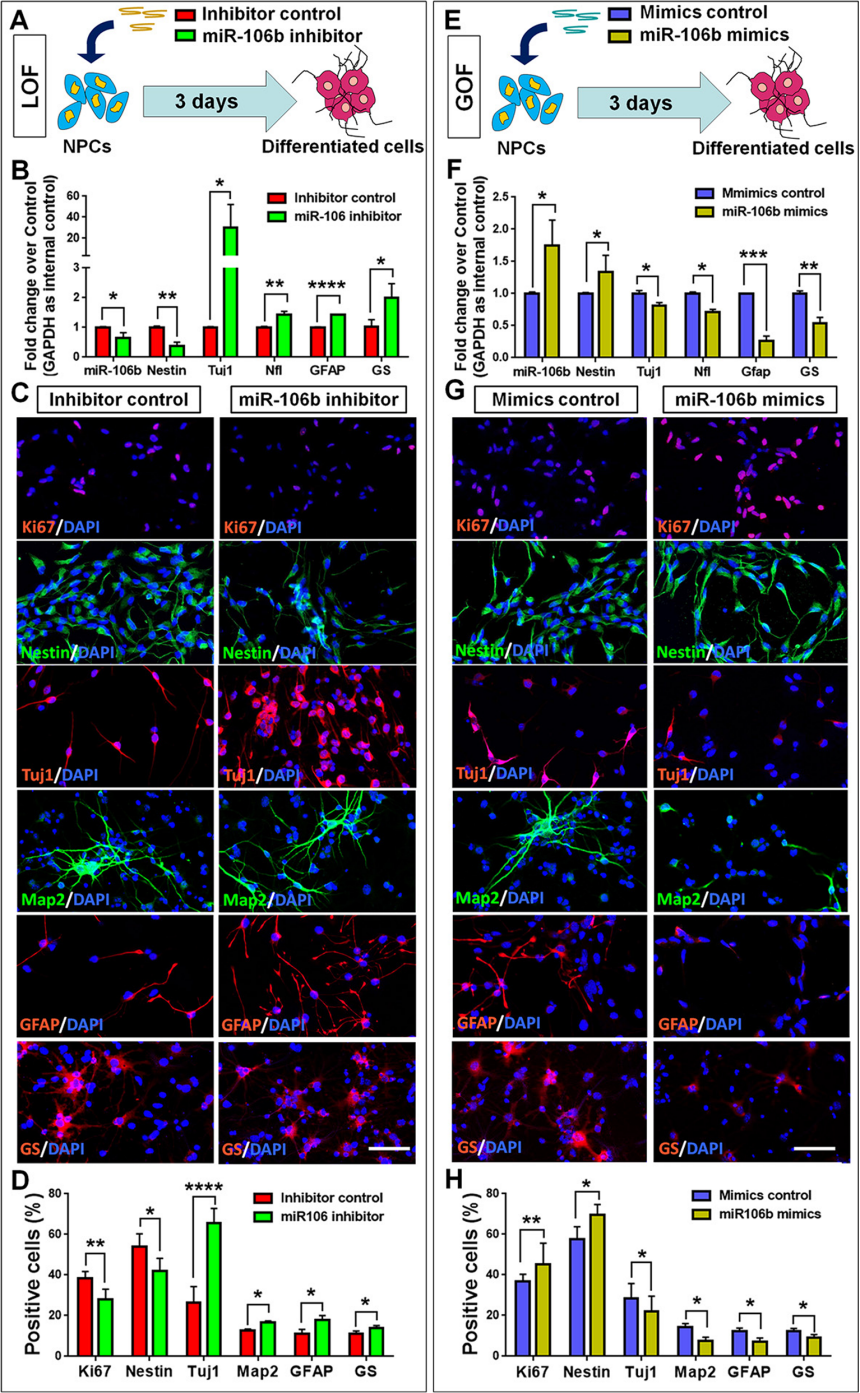
*miR-106b* inhibits the differentiation of NPCs. **a** A schematic representation of *miR-106b* LOF approach. **b** qPCR analysis of expression levels of *miR-106b* and transcripts corresponding to markers of NPCs (*Nestin*) and differentiated cells (*Tuj1*/*GFAP*) in the *miR-106b* LOF groups, compared to controls. **c** Immunofluorescence analysis of transduced cells displaying proliferating NPC (Ki67/Nestin)- and differentiated cell (Tuj1/GFAP)-specific immunoreactivities. **d** Quantification of cells displaying immunoreactivities corresponding to proliferating NPCs and differentiated cells in the *miR-106b* LOF group, compared to controls. **e** A schematic representation of *miR-106b* GOF approach. **f** qPCR analysis of expression levels of *miR-106b* and transcripts corresponding to markers of NPCs (*Nestin*) and differentiated cells (*Tuj1*/*GFAP*) in the *miR-106b* GOF group, compared to controls. **g** Immunofluorescence analysis of transduced cells displaying proliferating NPC (Ki67/Nestin)- and differentiated cell (Tuj1/GFAP)-specific immunoreactivities. **h** Quantification of cells displaying immunoreactivities corresponding to proliferating NPCs and differentiated cells in the *miR-106b* GOF group, compared to controls. Scale bar, 50 μm (**c**, **g**). Data are mean ± SD. ∗∗∗∗*p* < 0.0001, ∗∗∗*p* < 0.001, ∗∗*p* < 0.01, and ∗*p* < 0.05. Experiments were carried out three times in triplicates for in vitro perturbation

Next, we examined the roles of *miR-106b* in the differentiation of NPCs. Similar to previous studies, we carried out LOF and GOF approaches to address the involvement of *miR-106b* in the differentiation conditions. NPCs were firstly transfected with either *miR-106b* inhibitor (=LOF group) or inhibitor control and cultured in differentiation conditions for 3 DIV (Fig. 3a). qRT-PCR analysis revealed a significant reduction of *miR-106b* levels, validating the transfection (Fig. 3b). We also observed downregulation of *Nestin* transcripts expression, together with the increase of expression levels of transcripts corresponding to neuronal- and glial-specific markers *Tuj1* and *GFAP*, respectively (Fig. 3b). qRT-PCR results were corroborated by immunofluorescence analysis, where the proportions of cells immunoreactive for Ki67 and Nestin were reduced significantly while that of cells immunoreactive for Tuj1 and GFAP were significantly increased, confirming that *miR-106b* LOF promoted the differentiation of NPCs (Fig. 3c, d).

**Fig. 4:**
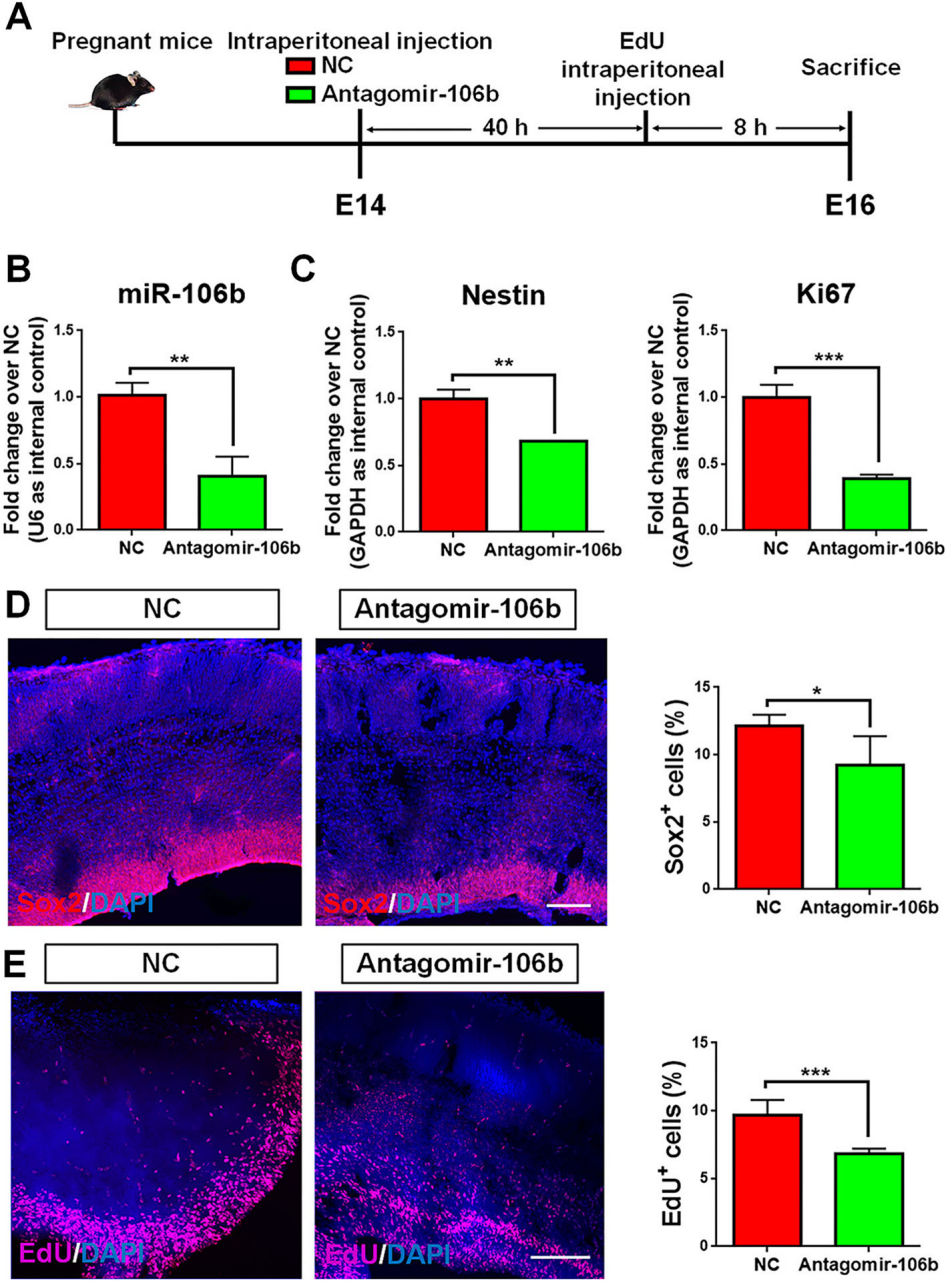
*miR-106b* regulates the maintenance of NPCs in vivo. **a** A schematic representation of *miR-106b* LOF approach in vivo. **b** qPCR analysis of expression levels of *miR-106b* in the *miR-106b* LOF and control groups, compared to their respective controls. **c** qPCR analysis of transcripts corresponding to markers of NPCs (*Nestin*) and proliferating cells (*Ki67*) in the *miR-106b* LOF and control groups. (**d**, **e**) Immunofluorescence analysis and quantification of transduced cells displaying Sox2- (**d**) and EdU (**e**)-specific immunoreactivities. Scale bar, 10 μm (**d**, **e**). Data are mean ± SD. ∗∗∗*p* < 0.001, ∗∗*p* < 0.01, and ∗*p* < 0.05. Experiments were carried out three times in triplicates with 5–7 E14 embryos per group three times in triplicates for in vivo perturbation

In the *miR-106b* GOF approach, NPCs were transfected with either *miR-106b* mimics or inhibitor control and cultured in differentiation conditions for 3 DIV (Fig. 3e). In contrast to the results obtained by the LOF approach, the ectopic expression of *miR-106b* significantly inhibited the differentiation of both neuronal and glial cells, as ascertained by significant decreases in levels of transcripts corresponding to cell type-specific markers and the number of cells displaying cell-type-specific immunoreactivities (Fig. 3f–h). Therefore, both LOF and GOF studies demonstrated that *miR-106b* negatively regulated the differentiation of NPCs, regardless of neuronal and glial lineages.

Our results revealed that the function of *miR-106b* is different from that of *miR-17* which regulates the neurogliogenic decision. To examine whether *miR-106b* paralog, *miR-106a*, has similar function, we tested the effects of *miR-106a* in NPCs’ differentiation using the same LOF and GOF approaches in differentiation conditions. The upregulation or downregulation of *miR-106a* levels in NPCs did not significantly regulate the differentiation of NPCs, confirmed by qRT-PCR and immunofluorescence analyses (Additional file 1: Figure S2). Hence, our observations indicated that *miR-106b*, but not *miR-106a*, significantly regulates the proliferation and differentiation of NPCs, suggesting that *miR-106b* may play a central role in the regulation of NPCs, compared with its paralogs.

### *miR-106b* does not regulate the neuronal subtype specification of NPCs

To further examine the influence of *miR-106b* on the cell fate determination of NPCs, especially on the neuronal subtype specification, we carried out long-term differentiation (14 DIV) of NPCs for sufficient cell fate commitment and maturation of differentiated cells under both *miR-106b* LOF and GOF conditions. Similar to our observations in short-term differentiation study, extended culture did not affect the commitment of neuronal and glial lineages. The generation of neurons and astrocytes was equally enhanced and inhibited in *miR-106b* LOF and GOF conditions, respectively (Additional file 1: Figure S3). The immunofluorescence analysis using neuronal subtype-specific antibodies corresponding to glutamatergic neurons (vGlut), GABAergic neurons (Gaba), and cholinergic neurons (ChAT) revealed that *miR-106b* had no preference in regulating the differentiation of neuronal subtypes, which was validated by the coincident patterns of the proportions of each cell type in both *miR-106b* LOF and GOF conditions (Additional file 1: Figure S3).

### *miR-106b* has no effects on the survival of NPCs

To examine the contribution of apoptosis in the effects of *miR-106* in NPCs’ regulation, we carried out terminal deoxynucleotidyl transferase dUTP nick end labeling (TUNEL) assay, detecting DNA fragmentation by labeling the terminal end of nucleic acids. The cell counting results revealed similar proportions of TUNEL^+^ cells either in *miR-106b* mimics- or in *miR-106b* inhibitor-treated groups, compared with controls, when NPCs were cultured in proliferation medium (Additional file 1: Figure S4A, B). Same results were observed during NPCs’ differentiation, that the modification of *miR-106b* expression levels did not affect the survival of differentiated cells (Additional file 1: Figure S4C, D). Similar results were obtained that there was no significant difference in the proportions of TUNEL^+^ cells either in *miR-106a* mimics- or in *miR-106a* inhibitor-treated groups versus controls in both proliferation and differentiation conditions (Additional file 1: Figure S5). Thus, our results suggested that both *miR-106b* and *miR-106a* had no significant effects on the survival of NPCs in both the proliferation and differentiation conditions.

### *miR-106b* regulates the maintenance of NPC pool in vivo

Our in vitro studies suggested the importance of *miR-106b* in the maintenance of NPCs’ proliferation and stemness. To further validate our observations, we carried out *miR-106b* knockdown in E14 C57BL/6J mice (Fig. 4a). Either negative control (NC) or antagomir-106b (*=*LOF group) was administrated into pregnant mice intraperitoneally, and animals were sacrificed after 48 h. The expression of *miR-106b* in the cortex of E14 mice was repressed in the *miR-106b* LOF group, versus controls, indicating the successful knockdown of *miR-106b* in vivo (Fig. 4b). qPCR results revealed a significant reduction for the expression of transcripts corresponding to *Nestin* and *Ki67*, in the *miR-106b* LOF group, compared to controls (Fig. 4c). The *miR-106b* LOF group also exhibited a significant decrease in the proportions of cells displaying Sox2 and EdU immunoreactivities, when compared to NC group (Fig. 4d, e). Besides, the *miR-106b* LOF enhanced the differentiation of NPCs, ascertained by the increase of *Tuj1* and *GS* transcripts in the *miR-106b* LOF group, versus controls (Additional file 1: Figure S6). Thus, our results suggested that *miR-106b* plays an important role in the maintenance of NPC pool in vivo.

### Tp53inp1-Tp53-Cdkn1a axis is a target of *miR-106b*

**Fig. 5:**
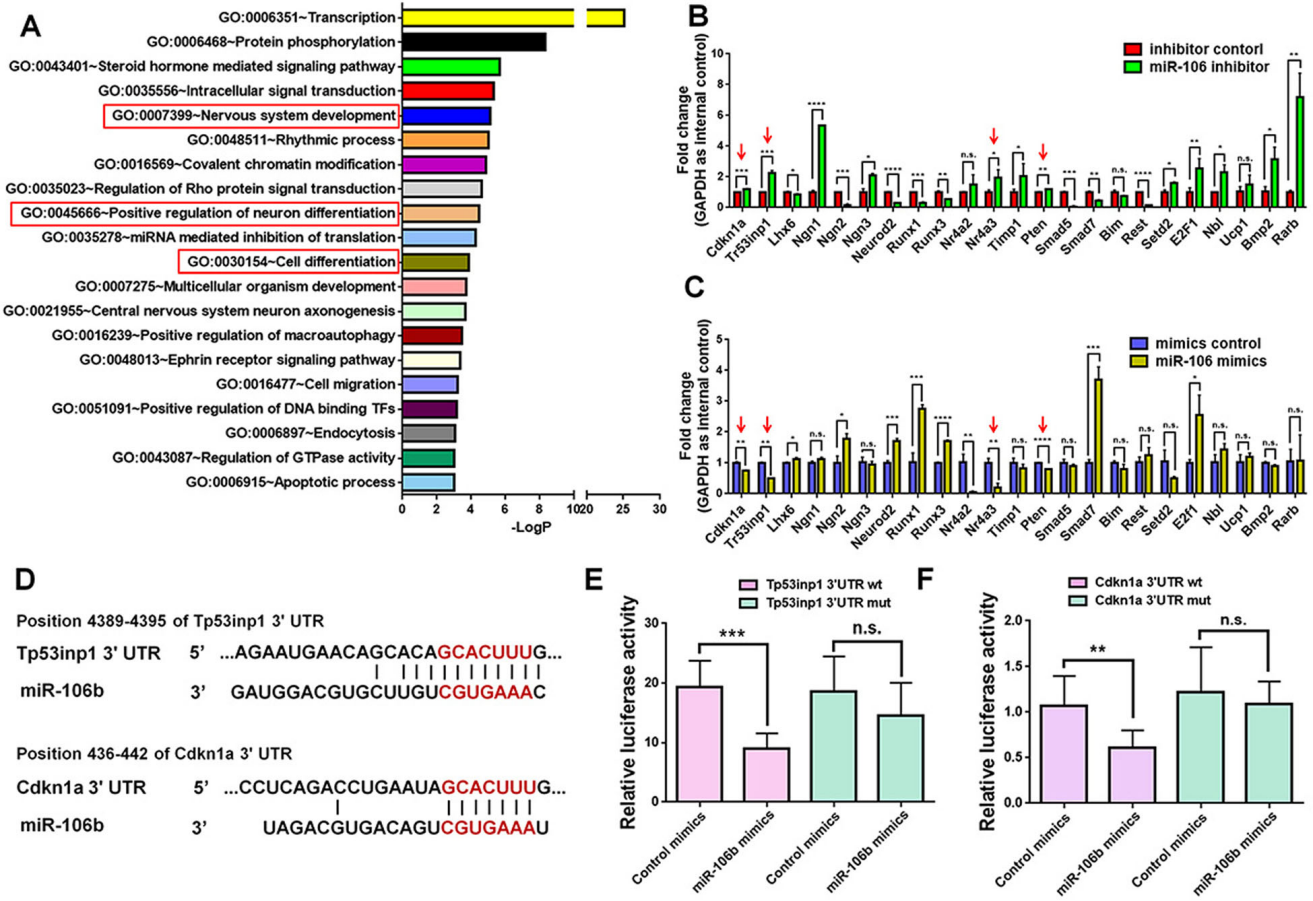
*miR-106b* targets Tp53inp1-Tp53-Cdkn1a axis in NPCs. **a** The GO analysis of top 20 enriched biological processes in the predicted *miR-106b* target genes. **b**, **c** qPCR analysis of candidate *miR-106b* target genes in the *miR-106b* LOF (**b**) and GOF (**c**) groups, compared to their respective controls. **d** The predicted consequential pairing of 3′UTR of candidate genes (top) and *miR-106b* (bottom) on the TargetScan website. **e**, **f** Repression of luciferase activities by the Tp53inp1 (**e**) and Cdkn1a (**f**) 3′UTR were dependent on *miR-106b*. Firefly luciferase activities were normalized to the internal control, Renilla luciferase activities. Data are mean ± SD. ∗∗∗∗*p* < 0.0001, ∗∗∗*p* < 0.001, ∗∗*p* < 0.01, and ∗*p* < 0.05. Experiments were carried out three times in triplicates for in vitro perturbation

To investigate the underlying mechanisms of *miR-106b*-mediated regulation of NPCs, we firstly extracted the predicted targets of *miR-106b* from Targetscan.org, a miRNA target prediction database. Over 900 transcripts exhibited conserved *miR-106b* target sites on their 3′UTR. In order to identify the putative candidates of *miR-106b*, Gene Ontology (GO) analysis of the predicted targets was carried out. The highest enriched GO terms, sorted out by the DAVID bioinformatics platform and Panther Classification System based on the biological function of genes, demonstrated the predicted targets of *miR-106b* were abundantly clustered into the categories of Nervous system development (GO: 0007399), Positive regulation of neuron differentiation (GO: 0045666), and Cell differentiation (GO: 0030154) (Fig. 5a).

To select putative targets of *miR-106b*, we filtered out 23 genes, which were reported to regulate the proliferation or differentiation of stem cells, in above three GO terms. The qPCR analysis of these genes demonstrated that the expression levels of the transcripts corresponding to *Tp53inp1*, *Cdkn1a*, *Nr4a3*, and *Pten* were inversely correlated with that of *miR-106b* in both *miR-106b* LOF and GOF approaches in the proliferation conditions (Fig. 5b, c).

Recently, the Tp53inp1-Tp53-Cdkn1a axis was shown to regulate multiple cellular activities including proliferation and survival of tumor cells [21, 22]. Interestingly, two factors in the axis have *miR-106b* target sites, and their expression was negatively regulated by *miR-106b*, revealing this axis as potential key downstream pathways of *miR-106b*-mediated effects on NPCs. To confirm the interaction between *miR-106b* and *Tp53inp1*/*Cdkn1a*, Luciferase assay was carried out (Fig. 5d–f). Co-transfection of *miR-106b* mimics and Dual-Luciferase reporter constructs containing the wild-type 3′UTR of *Tp53inp1* and *Cdkn1a*, but not *miR-106b* target site mutated 3′UTR of *Tp53inp1* and *Cdkn1a*, significantly decreased the firefly activity in HEK293A cells, normalized by the Rellina activity, indicating *miR-106b* directly targets *Tp53inp1* (Fig. 5e) and *Cdkn1a* (Fig. 5f).

### *miR-106b* regulates the proliferation and differentiation of NPCs through Tp53inp1-Tp53-Cdkn1a axis

Since *miR-106b* directly targeted and regulated the expression of Tp53inp1 and Cdkn1a, we hypothesized that *miR-106b* might influence the proliferation and differentiation of NPCs through Tp53inp1-Tp53-Cdkn1a axis. To test our premise, we knocked down the expression of *Tp53inp1* and *Cdkn1a* using siRNAs after *miR-106b* inhibitor treatment, where both *Tp53inp1* and *Cdkn1a* expression was significantly upregulated. Four siRNAs targeting different sites of transcripts corresponding to *Tp53inp1* and *Cdkn1a* were transfected into NPCs, and the siRNA silencing efficiency was examined by qPCR analysis 72 h post-transfection (Additional file 1: Figure S7). siRNAs showing the highest silencing efficiency were selected for the following studies.

First, we examined the roles of Tp53inp1 and Cdkn1a in the proliferation and differentiation of NPCs. The knockdown of either Tp53inp1 or Cdkn1a by siRNA significantly increased the numbers and sizes of neurospheres generated by NPCs, compared with controls (Additional file 1: Figure S8A-C, H-J). qRT-PCR analysis and immunocytochemical analyses revealed that either Tp53inp1 or Cdkn1a siRNA treatment significantly elevated the expression of Ki67, Nestin, and Sox2 in both transcripts and protein levels (Additional file 1: Figure S8D-G, K-N). Moreover, the Tp53inp1 and Cdkn1a siRNA treatment preserved the stemness of NPCs and significantly downregulated the differentiation capacity of NPCs into both neuronal and glial lineages, by examining the transcript and protein expression of markers for NPCs and differentiated cells through qRT-PCR and immunofluorescence analyses (Additional file 1: Figure S9). No significant effects were observed for Tp53inp1 and Cdkn1a on the survival of NPCs in both siRNA-treated groups versus controls (Additional file 1: Figure S10). Thus, in contrast with the effects of *miR-106b*, both Tp53inp1 and Cdkn1a inhibit the proliferation and accelerate the differentiation of NPCs.

**Fig. 6:**
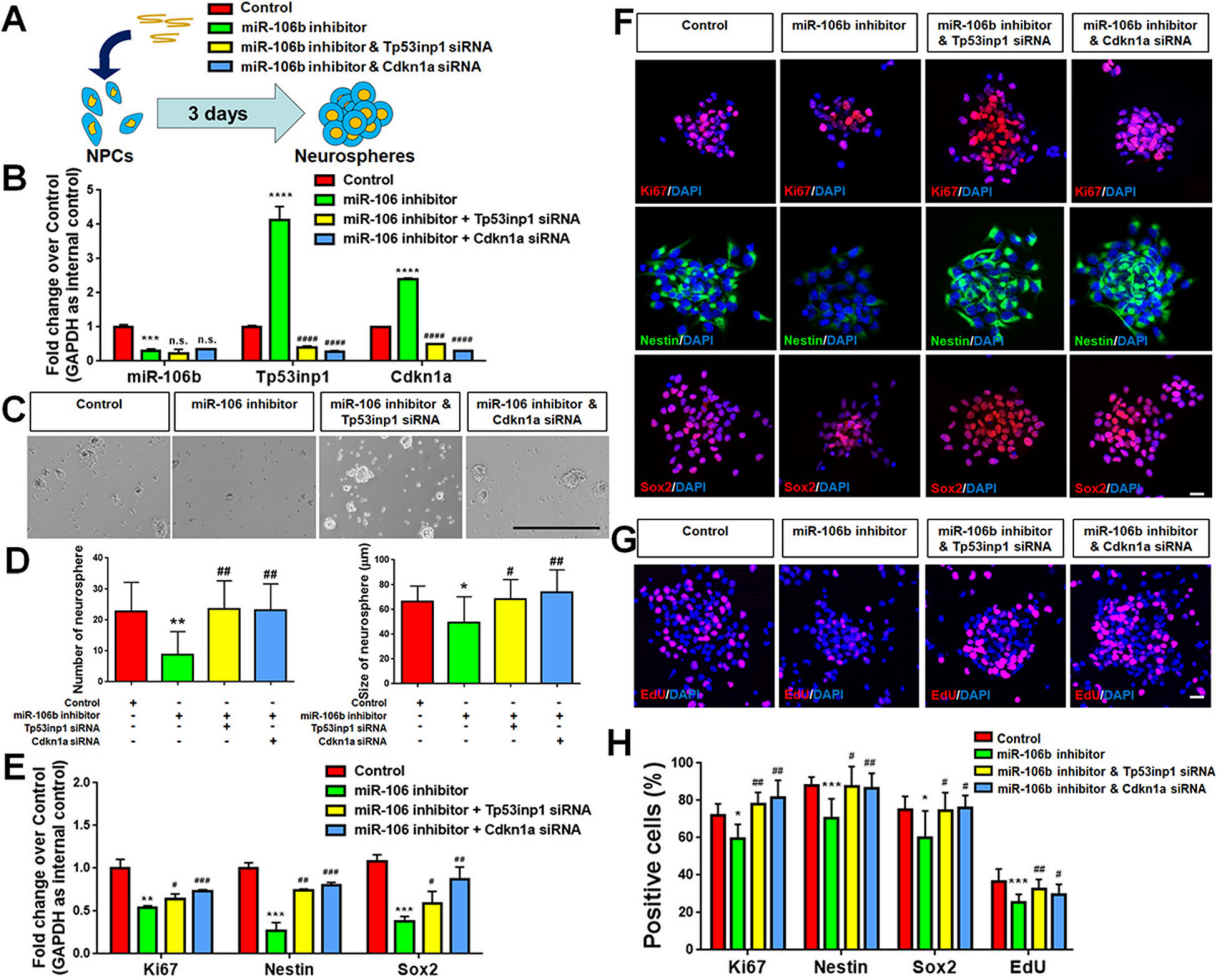
**a**–**h** *miR-106b* promotes the proliferation of NPCs through Tp53inp1-Tp53-Cdkn1a axis. **a** A schematic representation of the experimental approach. **b** qPCR analysis of expression levels of *miR-106b* and transcripts corresponding to *Tp53inp1* and *Cdkn1a* in all experimental groups versus controls. **c**, **d** The number and size of neurospheres were quantified in all experimental groups versus controls. **e** qPCR analysis of levels of *miR-106b* and transcripts corresponding to *Ki67*, *Nestin*, *Sox2*, *Tp53inp1*, and *Cdkn1a* in all experimental groups versus controls. **f** Immunofluorescence analysis of transduced cells displaying proliferating cells (Ki67)- and NPCs (Nestin/Sox2)-specific immunoreactivities in all experimental groups versus controls. **g** Immunofluorescence analysis of transduced cells displaying EdU immunoreactivities in all experimental groups versus controls. **h** Quantification of cells expressing Ki67/Nestin/Sox2/EdU immunoreactivities in all experimental groups versus controls. Scale bar, 400 μm (**b**) and 20 μm (**e**, **f**). Data are mean ± SD. ∗∗∗∗*p* < 0.0001, ∗∗∗*p* < 0.001, ∗∗*p* < 0.01, and ∗*p* < 0.05. ^####^*p* < 0.0001, ^*###*^*p* < 0.001, ^##^*p* < 0.01, and ^#^*p* < 0.05 versus the *miR-106b* LOF groups. Experiments were carried out three times in triplicates for in vitro perturbation

Second, we examined the roles of Tp53inp1 and Cdkn1a in the *miR-106b*-mediated regulation of NPCs’ proliferation (Fig. 6a). NPCs were divided into 4 groups based on the inhibitor and siRNA transfection: control group (inhibitor control + siRNA control), *miR-106b* LOF group (*miR-106b* inhibitor + siRNA control), *miR-106b* and Tp53inp1 LOF group (*miR-106b* inhibitor + Tp53inp1 siRNA), and *miR-106b* and Cdkn1a LOF group (*miR-106b* inhibitor + Cdkn1a siRNA). The transfected cells were cultured in proliferation condition for 3 DIV to generate neurospheres. The LOF of Tp53inp1 and Cdkn1a was validated by qPCR analysis (Fig. 6b). The negative influence of *miR-106b* knockdown on the number and size of neurospheres was abrogated in both Tp53inp1 and Cdkn1a LOF groups (Fig. 6c, d). We also observed a significant increase in the expression of transcripts corresponding to *Ki67*, *Nestin*, and *Sox2* in Tp53inp1 and Cdkn1a LOF groups, versus *miR-106b* LOF group, suggesting the restorative effects of Tp53inp1 and Cdkn1a LOF on the proliferative capacity and stem cells properties of NPCs in *miR-106b* knockdown conditions (Fig. 6e). Our observations were corroborated with immunocytochemical analysis that the significant reduction of proportion of cells expressing immunoreactivities corresponding to Ki67, Nestin, Sox2, and EdU in *miR-106b* LOF groups was compromised by the silencing of *Tp53inp1* and *Cdkn1a* expression (Fig. 6f–h). Thus, our results suggested Tp53inp1 and Cdkn1a as key downstream effectors of *miR-106b* in the regulation of NPCs’ proliferation.

**Fig. 7:**
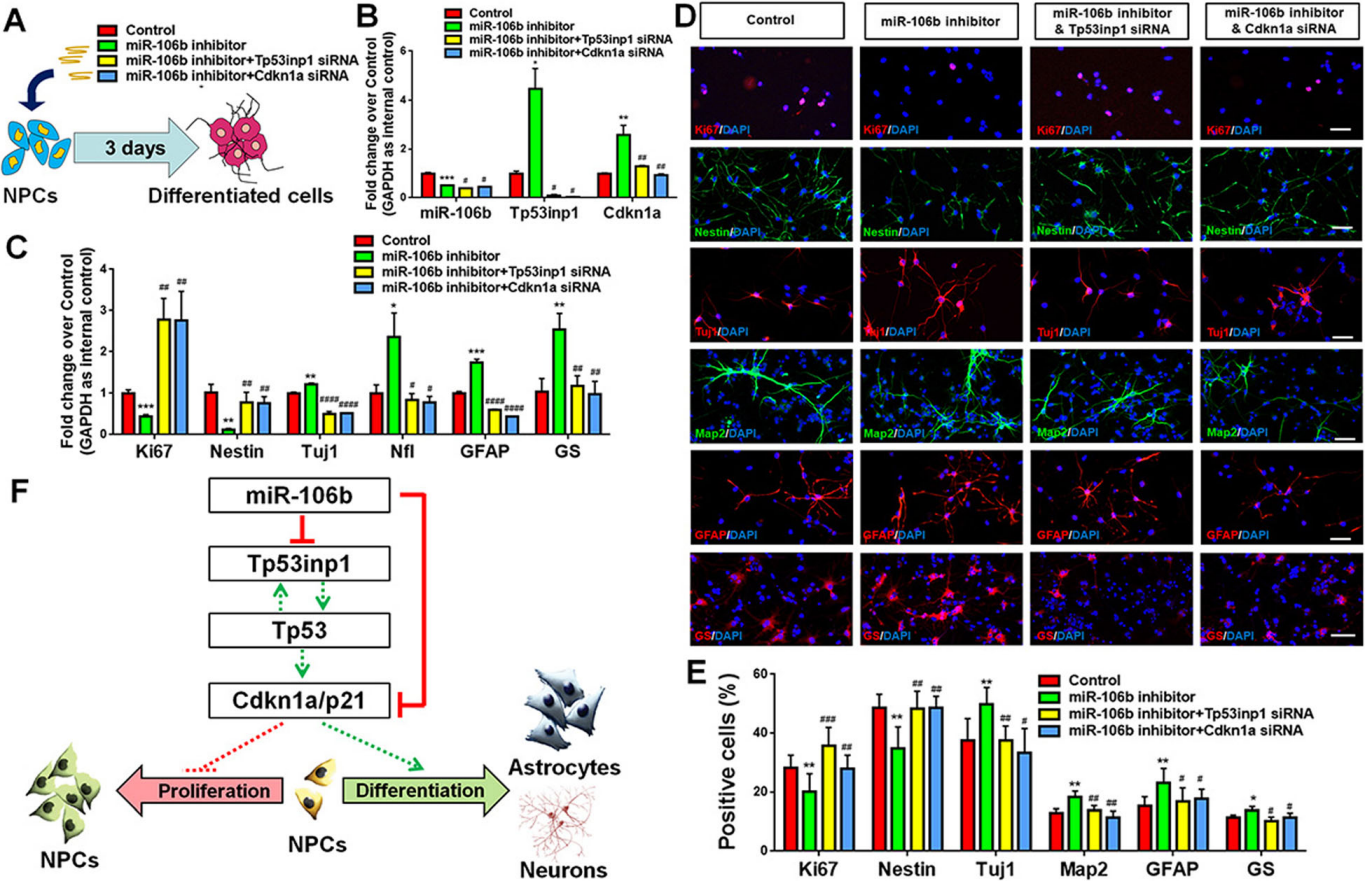
*miR-106b* inhibits the differentiation of NPCs through Tp53inp1-Tp53-Cdkn1a axis. **a** A schematic representation of the experimental approach. **b** qPCR analysis of levels of *miR-106b* and transcripts corresponding to *Tp53inp1* and *Cdkn1a* in all experimental groups versus controls. **c** qPCR analysis of levels of transcripts corresponding to *Ki67*, *Tuj1*, and *GFAP* in all experimental groups versus controls. **d** Immunofluorescence analysis of transduced cells displaying Ki67/Nestin and Tuj1/GFAP immunoreactivities in all experimental groups versus controls. **e** Quantification of cells expressing Ki67/Nestin and Tuj1/GFAP immunoreactivities in all experimental groups versus controls. **f** A schematic representation of *miR-106b*-mediated regulation of NPCs to facilitate proliferation: Tp53inp1-Tp53-Cdkn1a axis is a key inducer for cell cycle exit and differentiation of NPCs. The high levels of *miR-106b* inhibit Tp53inp1 and Cdkn1a expression, which maintains the proliferation of NPCs. The downregulation of *miR-106b* expression activates Tp53inp1-Tp53-Cdkn1a axis, leading to the decline of NPCs pool and the generation of both neurons and glia. Scale bar, 20 μm (**d**). Data are mean ± SD. ∗∗∗∗*p* < 0.0001, ∗∗∗*p* < 0.001, ∗∗*p* < 0.01, and ∗*p* < 0.05. ^####^*p* < 0.0001, ^###^*p* < 0.001, ^##^*p* < 0.01, and ^#^*p* < 0.05 versus the *miR-106b* LOF groups. Experiments were carried out three times in triplicates for in vitro perturbation

Third, we tested the roles of Tp53inp1 and Cdkn1a in the *miR-106b*-mediated regulation of NPCs’ differentiation. NPCs were divided into four groups using the same group setting in the proliferation study and cultured in differentiation conditions for 3 DIV (Fig. 7a). The transcript levels of *Tp53inp1* and *Cdkn1a* were significantly reduced in siRNA treatment groups, versus controls, validating the transfection efficiency (Fig. 7b). Moreover, the inhibitory effects of *miR-106b* knockdown in the expression of transcripts corresponding to Nestin, Tuj1, and GFAP were compromised by the Tp53inp1 and Cdkn1a LOF (Fig. 7c). The restoration of NPCs’ differentiation was confirmed by the immunocytochemical analysis by examining the proportion of cells displaying immunoreactivities corresponding to NPCs (Ki67/Nestin), neuronal (Tuj1/Map 2), and glial (GFAP/GS) markers (Fig. 7d, e). We observed that the positive effects of *miR-106b* LOF on the differentiation of both neuronal cell and glia were abrogated by either Tp53inp1 or Cdkn1a LOF. Besides, the reduction of Ki67^+^/Nestin^+^ cells in the *miR-106b* LOF group was restored by either Tp53inp1 or Cdkn1a LOF. Hence, our observation suggested that *miR-106b* regulates the proliferation and differentiation of NPCs via Tp53inp1 and Cdkn1a.

## Discussion

The development of the vertebrate CNS is a highly conserved dynamic process that involves progression through distinct stages, starting from the formation of neural tube [23, 24]. Neurogenesis, especially the maintenance and differentiation of NPCs, plays a central role in all these stages [25]. The impairment of neurogenesis during CNS development can cause severe developmental defects, such as malformations and amentia [24]. Identification of key factors in the regulation of neurogenesis is essential to fully understand the CNS development, which will benefit from preventing and treating developmental defects. Recently, *miR-17*~*106* family was reported to regulate neurogenesis of mouse developing/adult NPCs or ESC-derived NPCs [8, 15, 17]. Publications from multiple independent groups have shown that *miR-17*~*106* family serves as a cell cycle facilitator, mostly in cancer cells [10, 26, 27]. However, conflicting observations were also reported by Zhi et al. that *miR-106a* might negatively regulate proliferation through targeting *FASTK* in astrocytoma cells [28]. Besides the controversy for the effects of *miR-17*~*106* family on the proliferation of tumor cells, conflicting results were reported for that of *miR-17*~*106* family on the regulation of NPCs [8, 15]. In 2011, Brett et al. showed that *miR-106b*~*25* cluster can promote the neuronal differentiation of adult NPCs. However, in 2013, Bian et al. demonstrated the *miR-17*~*106* family inhibits the transition of NPCs to intermediate progenitors, which may block the generation of neuronal cells. Thus, these contradictory findings suggest two propositions: (1) the function of *miR-17*~*106* family, including *miR-106*, could be tissue- and cell type-specific in regulating proliferation and differentiation and (2) the roles of individual miRNAs in the family could be diverse although they may share similar seed sequence.

Here, we examined the involvement of *miR-106a*/*b* in the regulation of NPCs. We observed that, during brain development, the expression of *miR-106b* decreased with time, coincided with that of *Nestin* and *Ki67*. Similar expression patterns were also observed during NPCs’ differentiation in vitro. It is most likely that under the decrease of *miR-106b* expression, the maintenance of NPCs was attenuated, as demonstrated by a significant reduction of levels of transcripts corresponding to NPCs. The perturbation of function approaches also revealed that *miR-106b*, but not *miR-106a*, is important in the regulation of proliferation and differentiation of embryonic NPCs. It has been reported that individual component of miRNA families could exhibit distinct functions in the regulation of cell fate [17, 29]. Thus, our observations match with these reports that the effects of *miR-106a* and *miR-106b* on the regulation NPCs are different, even though they belong to the same family and share the same seed sequence.

In the in vitro studies, we demonstrated for the first time that, *miR-106b* positively and negatively regulated embryonic NPC’s proliferation and differentiation, respectively. When subjected to proliferation, NPCs’ phenotype was maintained by high levels of *miR-106b* and significant reduction of NPC pool was observed once *miR-106b* levels were downregulated. This result was matched with others’ findings that *miR-106b*, together with other *miR-17*~*106* family members, was tightly associated with the maintenance of NPCs [8, 15, 16]. When induced to differentiate, NPCs downregulated *miR-106b* and generated both neurons and glia. However, when the expression of *miR-106b* was perturbed, NPCs’ differentiation along neuronal and glial lineages was similarly compromised or enhanced. Our results suggested that *miR-106b* may serve as a proliferation “accelerator” and differentiation “break” and have no instructive effect in the commitment of certain lineages. It is highly possible that *miR-106b* may also be a key factor in regulating the balance of embryonic NPCs between proliferation and differentiation during brain development, due to the same positive correlation of the expression levels of *miR-106b* and the proportion of NPCs in vitro and in vivo, matching with Bian et al.’s observations.

The role of *miR-106b* in regulating proliferation and differentiation in extra-neural tissues is well known. As firstly investigated in cancer cells, *miR-106b*, together with all other *miR-17*~*106* family members, is considered as an oncogenic miRNA [10–13]. *miR-106b* is reported to promote the tumor growth through targeting tumor suppressor genes, such as *Pten*, *Cdkn1a, E2F1*, *Setd2*, *Runx3*, *Smad7*, *Cdkn1a*, and *Bim* [10, 13, 26, 30–33]. Besides, *miR-106b* negatively regulates the differentiation of various types of non-neural cells. For instance, *miR-106b* suppresses the differentiation of brown adipocytes and osteoblast through targeting *Ucp1* and *Smad5*, respectively [34, 35]. Thus, our observations matched with the situation in extra-neural tissues, suggesting a coincident function of *miR-106b* on diverse types of stem cells. Furthermore, our study identified Tp53inp1-Tp53-Cdkn1a axis as a key downstream regulatory network of *miR-106b*-mediated regulation of NPCs. Previously identified in cancer cells and mouse embryonic fibroblasts, Tp53inp1-Tp53-Cdkn1a axis is considered as a tumor suppressor pathway [21, 36, 37]. Tp53inp1, as a Tp53-induced nuclear protein, inhibits cell-cycle progression and promotes of apoptosis in a Tp53-dependent manner [21, 38]. Tp53inp1 also physically interacts with Tp53 and enhances its activity by Ser 46 phosphorylation, forming a positive feedback loop with Tp53 [21, 39]. The activation of Tp53inp1 and Tp53 leads to the elevation of Cdkn1a expression, resulting in cell-cycle arrest and proliferation reduction [40, 41]. In CNS, Tp53inp and Cdkn1a were also reported to regulate the expansion of postnatal and adult NPCs by regulating key genes for NPCs’ phenotype, such as Sox2 [16, 40, 42]. Hence, *miR-106b*-Tp53inp1-Tp53-Cdkn1a axis may act as an upstream pathway of Sox2-Lin28-*let-7* axis, an essential molecular mechanism for NPC proliferation and neurogenic potential, which forms a complete and comprehensive network on the regulation of NPCs [43].

## Conclusions

In summary, we demonstrated that *miR-106b* is highly expressed in embryonic NPCs. The decrease in *miR-106b* expression progressively shifts the balance toward NPCs’ differentiation. The mechanism involved is likely via the loss of *miR-106b*-mediated repression on the cell-cycle inhibitory network, Tp53inp-Tp53-Cdkn1a axis (Fig. 7f). Our findings demonstrate a unique master pathway on the regulation of NPCs, suggesting the necessity in exploring the exact roles and mechanisms underlying the effects of other miRNAs in *miR-17*~*106* family, which is currently under investigation.

## Additional file

Additional file 1: **Figure S1.** *miR-106a* does not promote the proliferation of NPCs. **Figure S2.** *miR-106a* does not regulate the differentiation of NPCs. **Figure S3.** *miR-106b* has no effect on neuronal subtype specification. **Figure S4.** *miR-106b* has no effect on NPCs’ survival. **Figure S5.** *miR-106a* has no effect on NPCs’ survival. **Figure S6.** *miR-106b* regulates the differentiation of NPCs in vivo. **Figure S7.** The validation of Tp53inp1 and Cdkn1a siRNAs. **Figure S8.** Tp53inp1 and Cdkn1a siRNAs promote the proliferation of NPCs. **Figure S9.** Tp53inp1 and Cdkn1a siRNAs inhibit the differentiation of NPCs. **Figure S10.** Tp53inp1 and Cdkn1a have no effect on NPCs’ survival. **Table S1.** List of specific primers. **Table S2.** List of primary antibodies. (DOCX 4511 kb)

## Data Availability

Data could be accessed through emails with the corresponding authors.

## Abbreviations

CNS: Central nervous system
DIV: Days in vitro
GOF: Gain-of-function
LOF: Loss-of-function
miRNA: MicroRNA
NPCs: Neural stem/progenitor cells
PFA: Paraformaldehyde
RT: Room temperature
