# Supplementary material for: miR-106b regulates the proliferation and differentiation of neural stem/progenitor cells through Tp53inp1-Tp53-Cdkn1a axis
Source: Stem Cell Res Ther. 2019 Sep 23;10:282. doi: 10.1186/s13287-019-1387-6 (PMC6755702; doi:10.1186/s13287-019-1387-6)

***miR-106b* regulates the proliferation and differentiation of neural stem/progenitor cells through Tp53inp1-p53-p21 axis**

Xiaohuan Xia, Chunhong Li, Hongfang Lu, Yunlong Huang, Yi Wang, Xiaoyu Yang, Jialin C. Zheng

**Supplemental Materials**

Figure S1

Figure S2

Figure S3

Figure S4

Figure S5

Figure S6

Figure S7

Figure S8

Figure S9

Figure S10

Table S1

Table S2

**
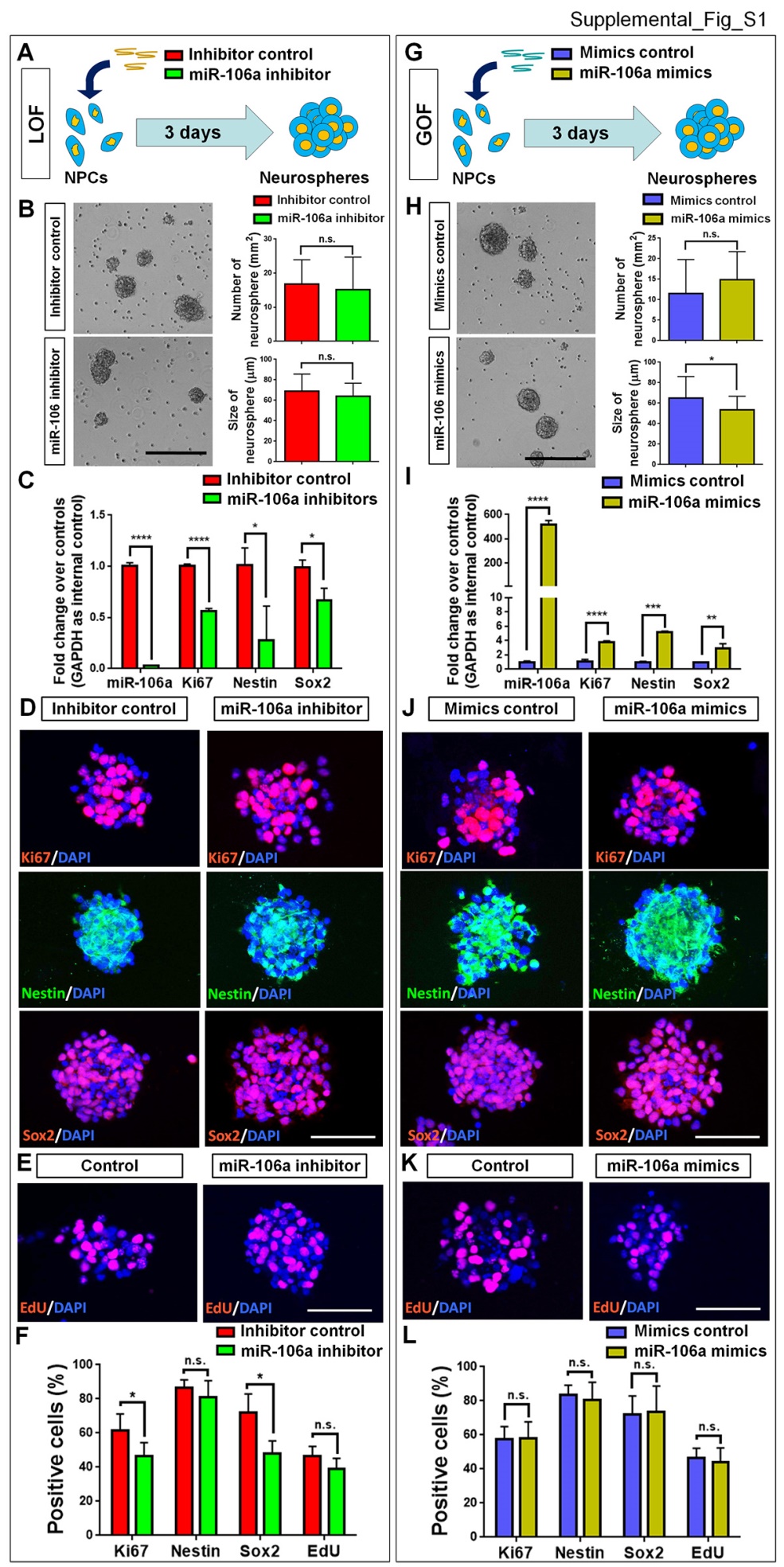
**

**Figure S1. *miR-106a* does not promote the proliferation of NPCs**.

(**A**) A schematic representation of *miR-106a* LOF approach; single NPCs were transduced with either *miR-106a* inhibitor or scrambled inhibitor control and subjected to proliferation for 3 DIV. (**B**) The number and size of neurospheres decreased in the *miR-106a* LOF group, compared to controls. (**C**) qPCR analysis of transduced cells revealed a significant decrease in levels of *miR-106b* and *Ki67*/*Nestin*/*Sox2* transcripts in *miR-106a* LOF group versus controls. (**D**) Immunofluorescence analysis of transduced cells after 3 DIV revealed no significant difference in proliferating cells- and NPCs-specific immunoreactivities in *miR-106a* LOF group, versus controls. (**E**) Immunofluorescence analysis of transduced cells after 3 DIV revealed no significant difference in EdU immunoreactivities in *miR-106b* LOF group, versus controls. (**F**) Quantification revealed no significant difference in the proportions of cells displaying immunoreactivities corresponding to proliferating cells (Ki67/EdU) and NPCs (Nestin/Sox2) in *miR-106a* LOF group, compared to controls. (**G**) A schematic representation of *miR-106a* GOF approach; single NPCs were transduced with either *miR-106a* mimics or scrambled mimics control and subjected to proliferation for 3 DIV. (**H**) The number and size of neurospheres increased in the *miR-106a* GOF group, compared to controls. (**I**) qPCR analysis of transduced cells revealed a significant increase in levels of *miR-106b* and *Ki67*/*Nestin*/*Sox2* transcripts in *miR-106a* GOF group versus controls. (**J**) Immunofluorescence analysis of transduced cells after 3 DIV revealed no significant difference in proliferating NPCs-specific immunoreactivities in *miR-106a* GOF group, versus controls. (**K**) Immunofluorescence analysis of transduced cells after 3 DIV revealed no significant difference in EdU immunoreactivities in *miR-106a* GOF group, versus controls. (**L**) Quantification revealed no significant difference in the proportions of cells displaying immunoreactivities corresponding to proliferating cells (Ki67/EdU) and NPCs (Nestin/Sox2) in *miR-106a* GOF group, compared to controls. Scale bar, 400 μm (B, H) and 50 μm (D, E, J, K). Amplification curves and gene expressions were normalized to the house-keeping gene GAPDH (for mRNA) and U6 snRNA (for miRNA). Data are mean ± sd. Experiments were carried out three times in triplicates for *in vitro* perturbation.

**
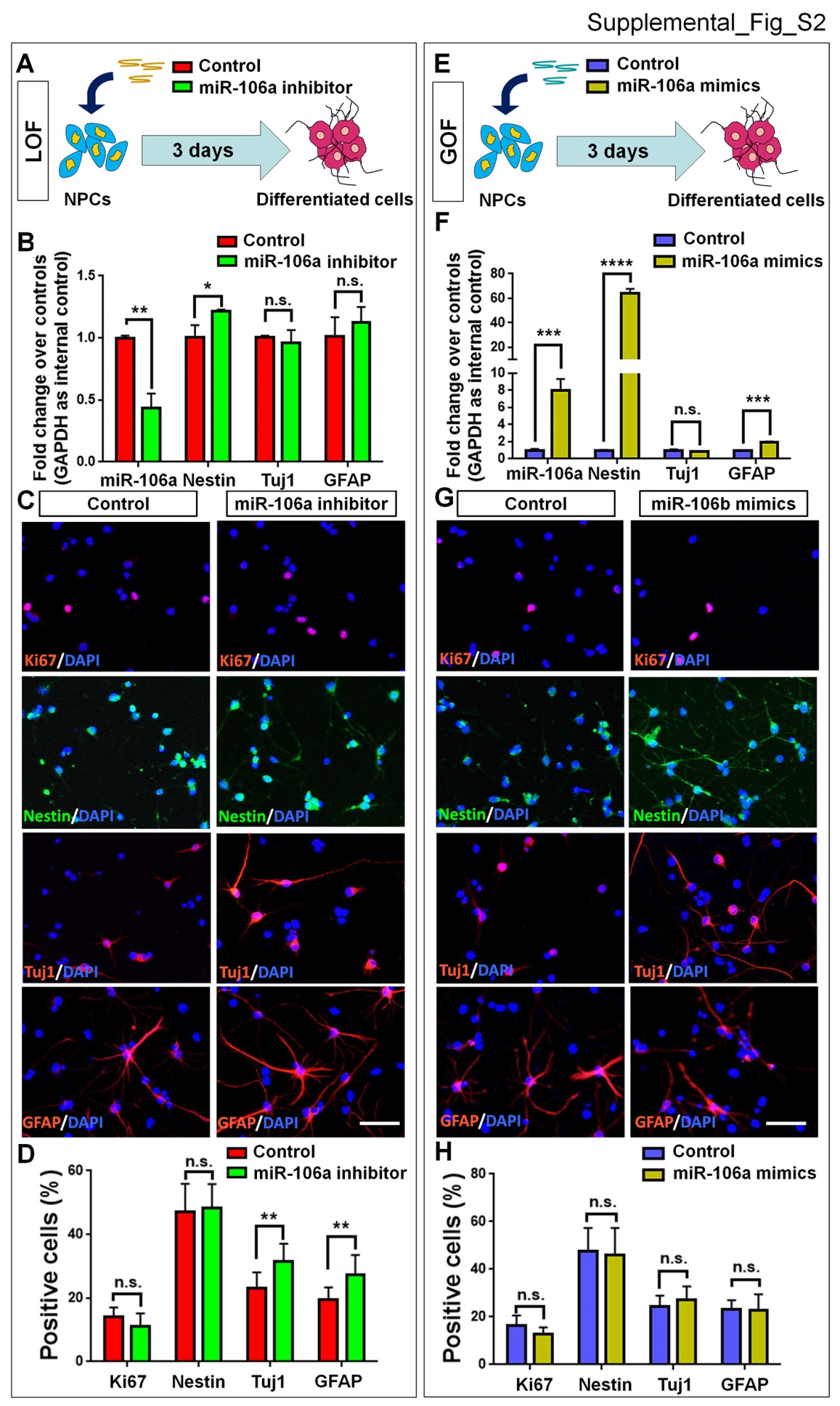
**

**Figure S2. *miR-106a* does not regulate the differentiation of NPCs.**

(**A**) A schematic representation of *miR-106a* LOF approach; single NPCs were transduced with either *miR-106a* inhibitor or scrambled inhibitor control and subjected to differentiation for 3 DIV. (**B**) Relative expression of *miR-106a* and transcripts corresponding to *Nestin*, *Tuj1* and *GFAP* after *miR-106a* inhibitor and scrambled inhibitor control transduction as assessed by qPCR. (**C**) Representative immunofluorescence with anti-Ki67, anti-Nestin, anti-Tuj1 and anti-GFAP for *miR-106a* inhibitor- or scrambled inhibitor control-transduced cells after 3 DIV. (**D**) Quantification revealed no significant difference and an significant increase in the proportions of cells expressing Ki67/Nestin and Tuj1/GFAP immunoreactivities, respectively, in *miR-106a* LOF group, compared to controls. (**E**) A schematic representation of *miR-106a* GOF approach; single NPCs were transduced with either *miR-106a* mimics or scrambled mimics control and subjected to differentiation for 3 DIV. (**F**) Relative expression of *miR-106a* and transcripts corresponding to *Nestin*, *Tuj1* and *GFAP* after *miR-106a* mimics and mimics inhibitor control transduction as assessed by qPCR. (**G**) Representative immunofluorescence with anti-Ki67, anti-Nestin, anti-Tuj1 and anti-GFAP for *miR-106a* inhibitor- or scrambled inhibitor control-transduced cells after 3 DIV. (**H**) Quantification revealed no significant difference in the proportions of cells displaying Ki67/Nestin and Tuj1/GFAP immunoreactivities, respectively, in *miR-106a* GOF group, compared to controls. Scale bar, 50 μm (C, G). Amplification curves and gene expressions were normalized to the house-keeping gene GAPDH (for mRNA) and U6 snRNA (for miRNA). Data are mean ± sd. Experiments were carried out three times in triplicates for *in vitro* perturbation.

**
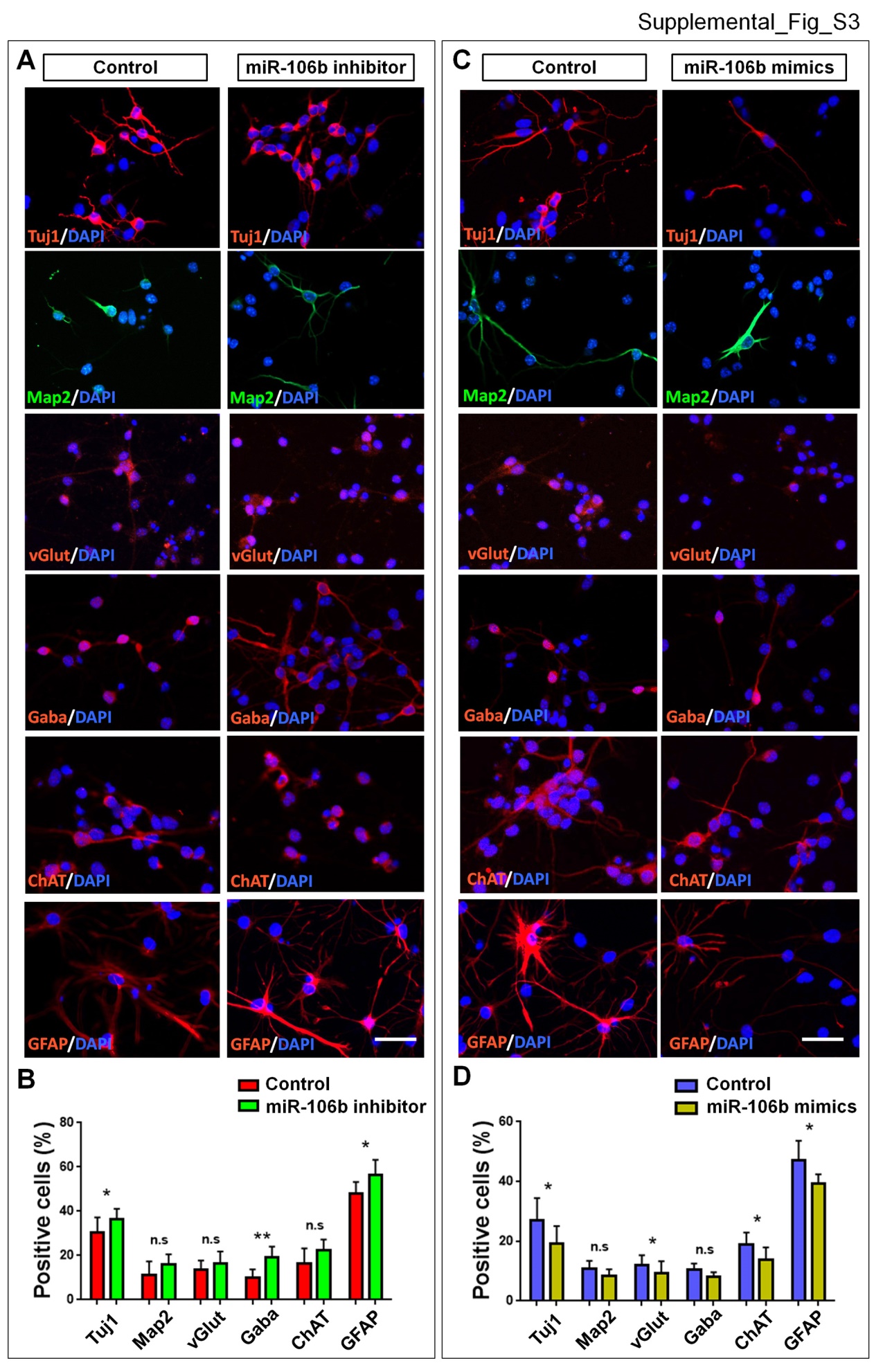
Figure S3. *miR-106b* has no effect on neuronal subtype specification.**

(**A**) Immunofluorescence analysis of transduced cells after 14 DIV displayed neuronal subtype-specific immunoreactivities in *miR-106b* LOF experiments. (**B**) Quantification revealed a significant increase in the proportions of cells expressing Tuj1, Gaba and GFAP immunoreactivities in the *miR-106b* LOF group, compared to controls. (**C**) Immunofluorescence analysis of transduced cells after 14 DIV displayed neuronal subtype-specific immunoreactivities in *miR-106b* GOF experiments. (**D**) Quantification revealed a significant decrease in the proportions of cells expressing Tuj1, vGlut, ChAT and GFAP immunoreactivities in the *miR-106b* GOF group, compared to controls. Scale bar, 20 μm (C, G). Data are mean ± sd. Experiments were carried out three times in triplicates for *in vitro* perturbation.

**
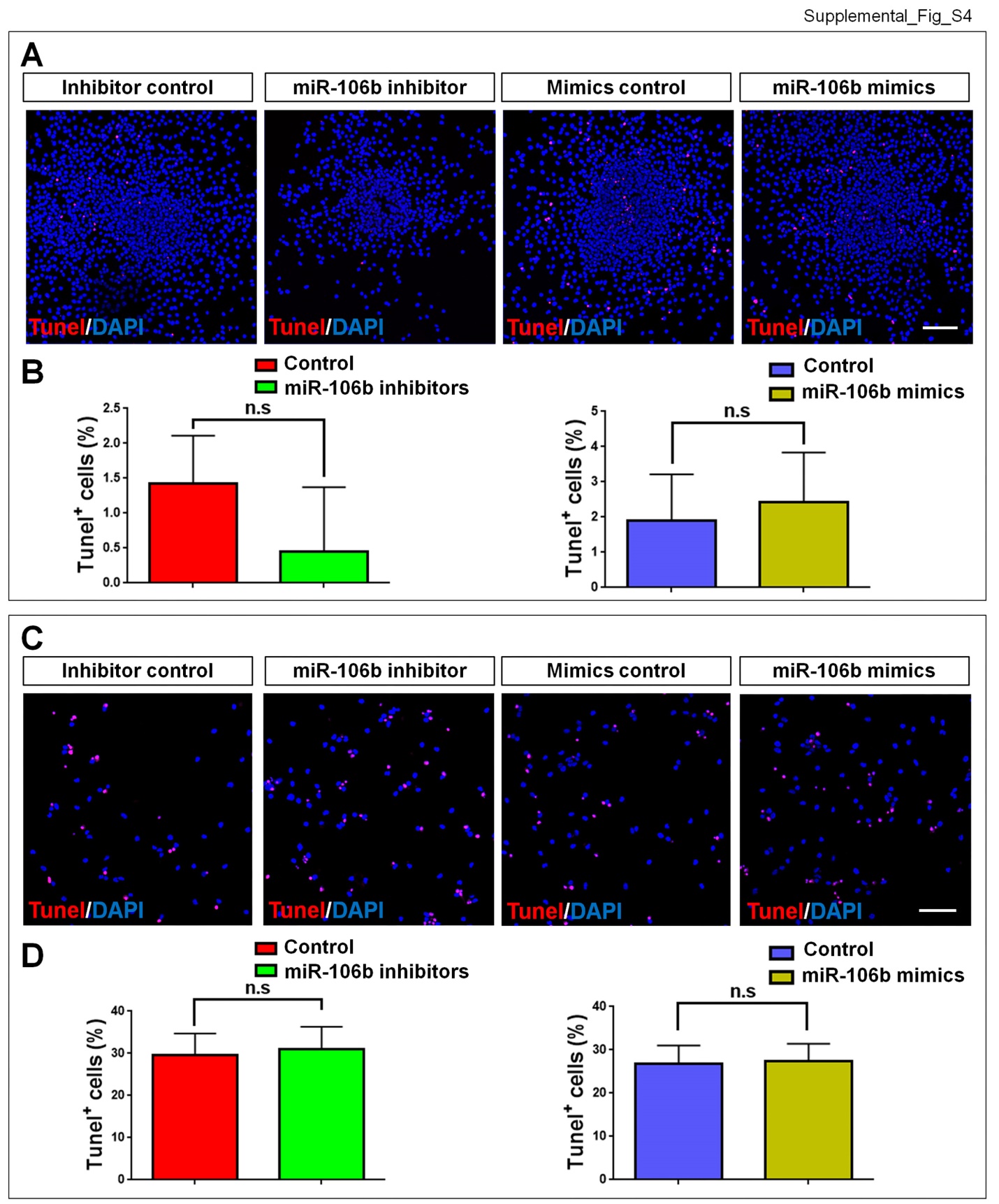
**

**Figure S4. *miR-106b* has no effect on NPCs survival.**

(**A**) Representative pictures of Tunel+ (red) cells in scrambled inhibitor control-, *miR-106b* inhibitor-, scrambled mimics control- and *miR-106b* mimics-transfected NPCs after 3DIV in proliferation conditions were shown. (**B**) Quantification revealed no significant difference in the proportions of Tunel+ cells in both *miR-106b* LOF and GOF group, compared to controls. (**C**) Representative pictures of Tunel+ (red) cells in scrambled inhibitor control-, *miR-106b* inhibitor-, scrambled mimics control- and *miR-106b* mimics-transfected NPCs after 3DIV in differentiation conditions were shown. (**D**) Quantification revealed no significant difference in the proportions of Tunel+ cells in both *miR-106b* LOF and GOF group, compared to controls. Scale bar, 50 μm (A, C). Data are mean ± sd. Experiments were carried out three times in triplicates for *in vitro* perturbation.

**
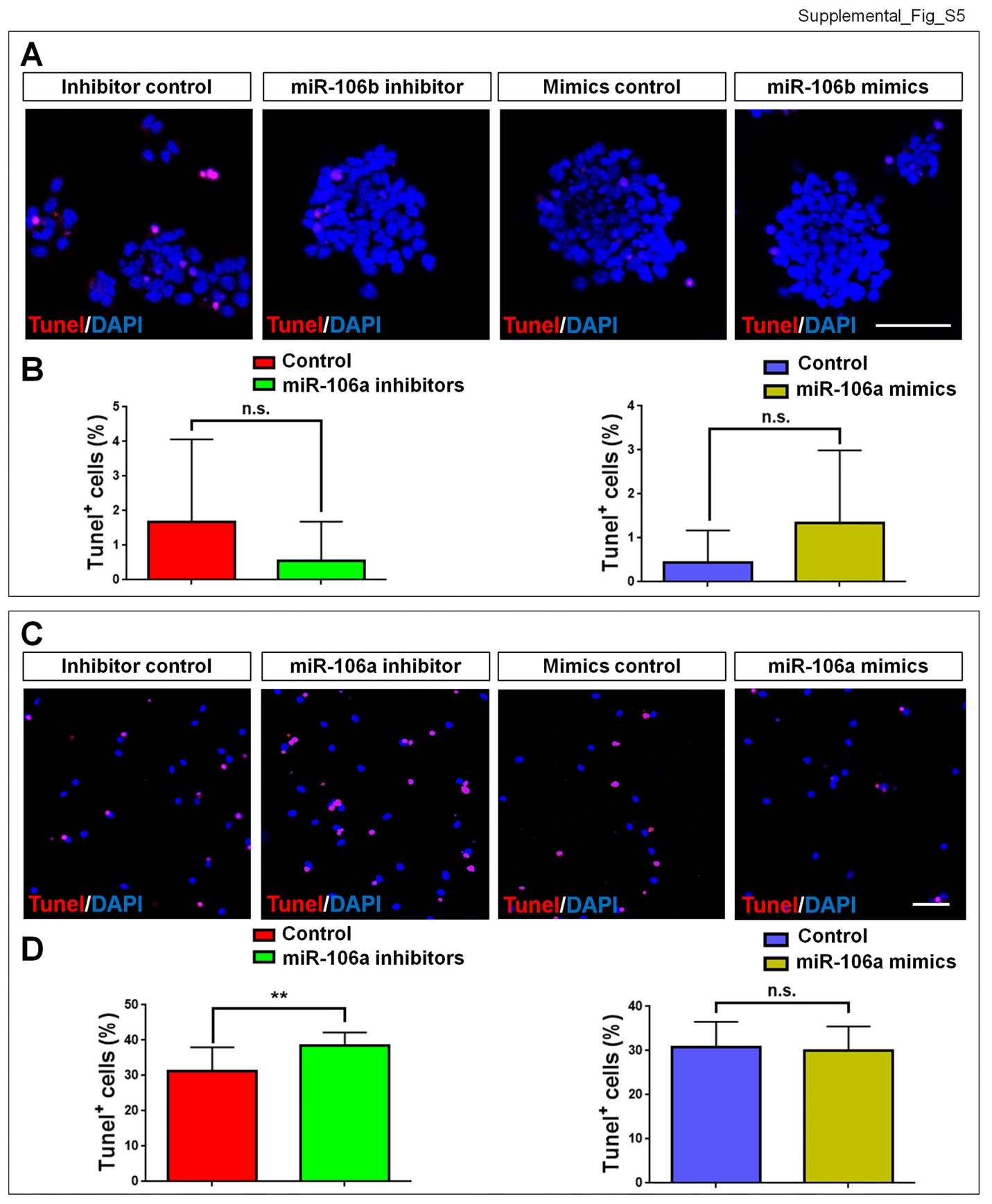
**

**Figure S5. *miR-106a* has no effect on NPCs survival.**

(**A**) Representative pictures of Tunel+ (red) cells in scrambled inhibitor control-, *miR-106a* inhibitor-, scrambled mimics control- and *miR-106a* mimics-transfected NPCs after 3DIV in proliferation conditions were shown. (**B**) Quantification revealed no significant difference in the proportions of Tunel+ cells in both *miR-106a* LOF and GOF group, compared to controls. (**C**) Representative pictures of Tunel+ (red) cells in scrambled inhibitor control-, *miR-106a* inhibitor-, scrambled mimics control- and *miR-106a* mimics-transfected NPCs after 3DIV in differentiation conditions were shown. (**D**) Quantification revealed no significant difference in the proportions of Tunel+ cells in both *miR-106a* LOF and GOF group, compared to controls. Scale bar, 50 μm (A, C). Data are mean ± sd. Experiments were carried out three times in triplicates for *in vitro* perturbation.


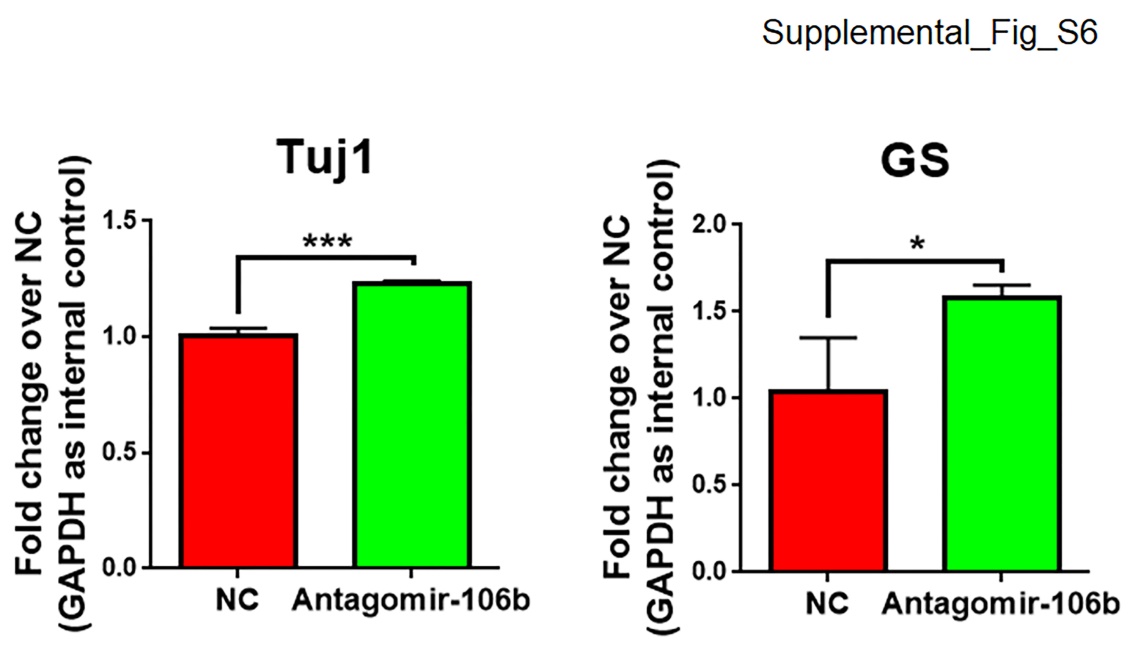


**Figure S6. *miR-106b* regulates the differentiation of NPCs *in vivo*.**

qPCR analysis of transcripts corresponding to neuronal (*Tuj1*) and glial (*GS*) markers in the *miR-106b* LOF and control groups.

**
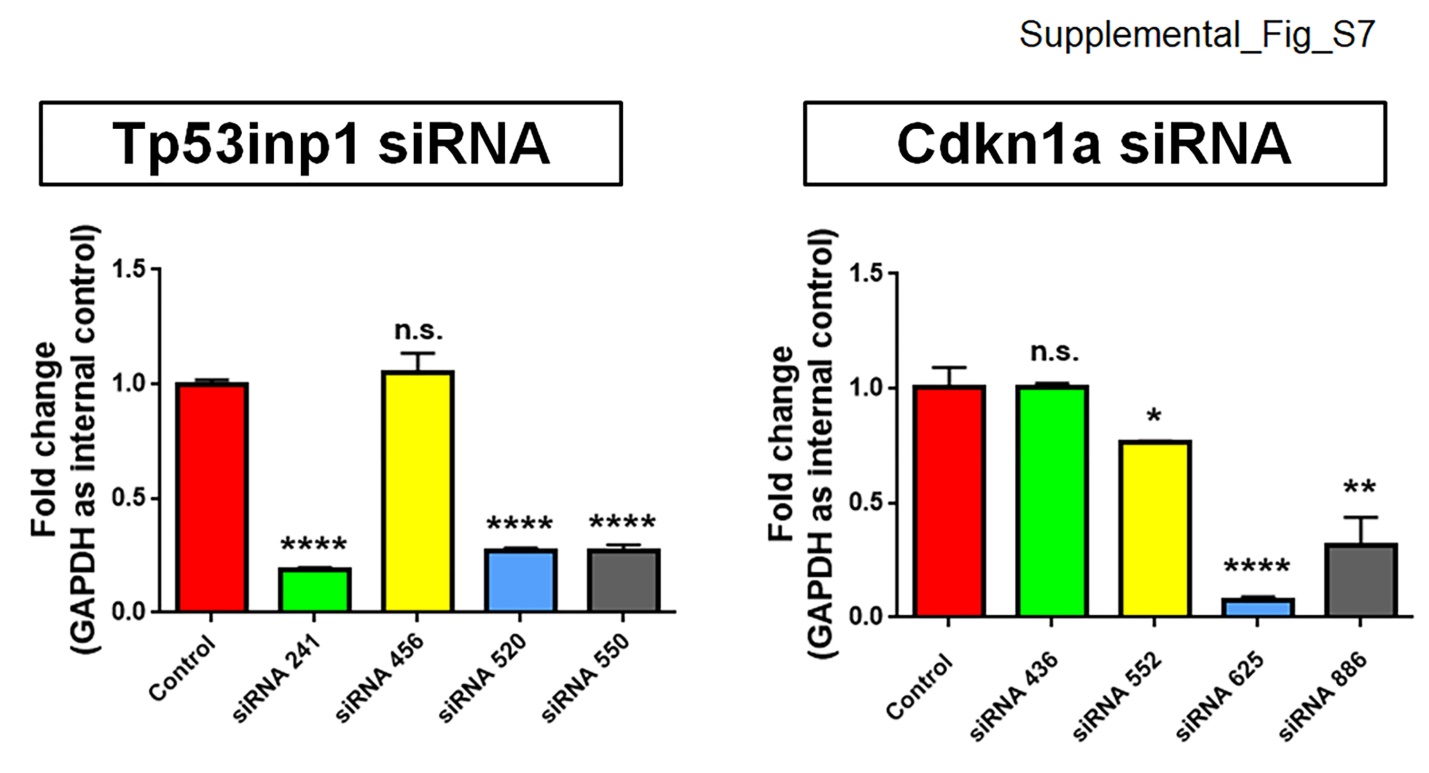
Figure S7. The validation of Tp53inp1 and Cdkn1a siRNAs.**

qPCR analysis of Tp53inp1 or Cdkn1a siRNAs-transduced cells was shown. 4 siRNAs for different target sites of Tp53inp1 and Cdkn1a were used for the silencing validation experiment. Amplification curves and gene expressions were normalized to the house-keeping gene GAPDH. Data are mean ± sd. Experiments were carried out three times in triplicates for in vitro perturbation.

**
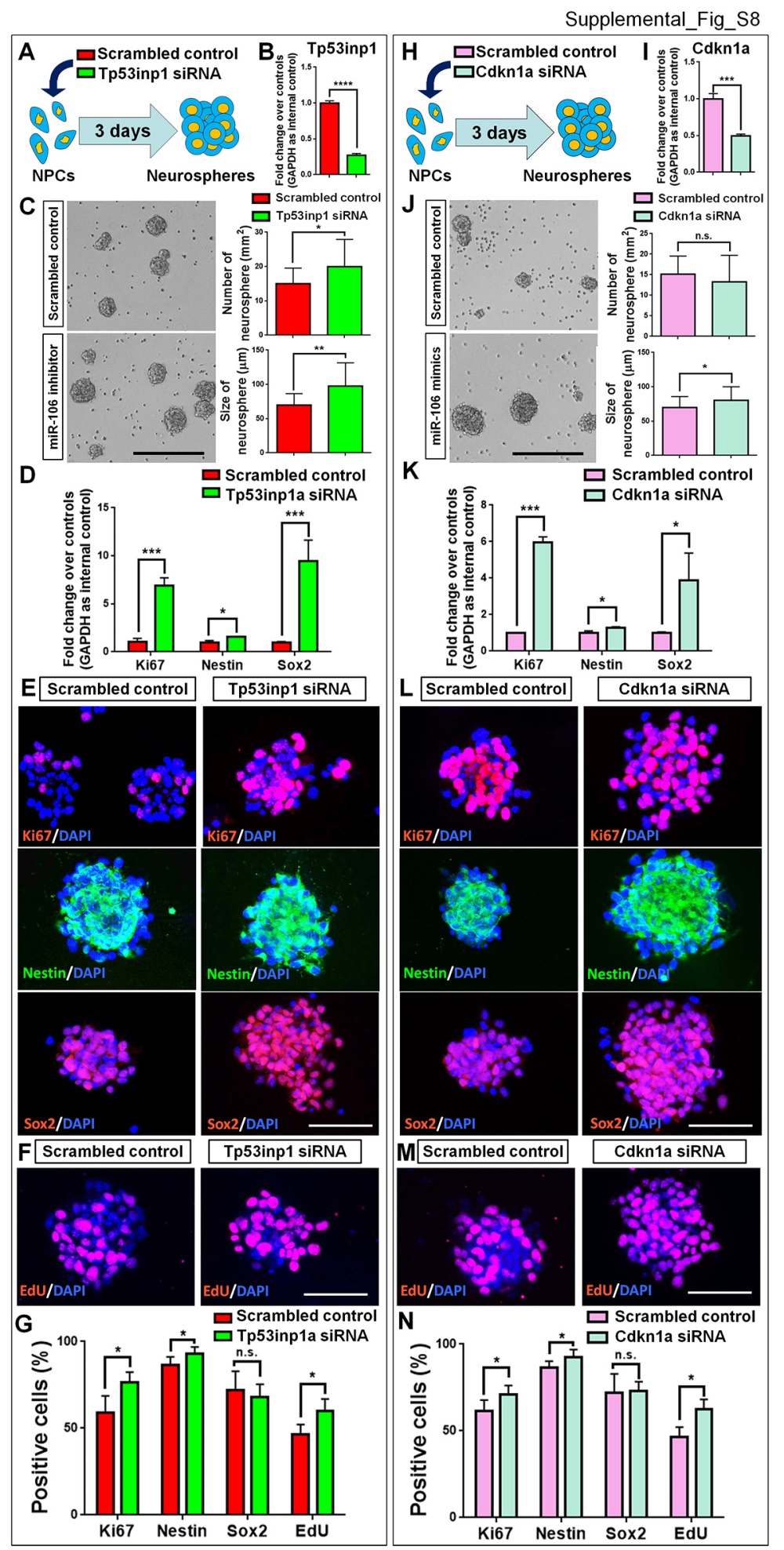
**

**Figure S8. Tp53inp1 and Cdkn1a siRNAs promote the proliferation of NPCs**.

(**A**) A schematic representation of Tp53inp1 LOF approach; single NPCs were transduced with either Tp53inp1 siRNA or scrambled control and subjected to proliferation for 3 DIV. (**B**) The transfection efficiency of Tp53inp1 siRNA was tested by qPCR. (**C**) The number and size of neurospheres increased in the Tp53inp1 LOF group, compared to controls. (**D**) qPCR analysis of transduced cells revealed a significant increase in levels of *Ki67*/*Nestin*/*Sox2* transcripts in Tp53inp1 LOF group versus controls. (**E**) Immunofluorescence analysis of transduced cells after 3 DIV revealed increase in proliferating cells- and NPCs-specific immunoreactivities in Tp53inp1 LOF group, versus controls. (**F**) Immunofluorescence analysis of transduced cells after 3 DIV revealed increase in EdU immunoreactivities in Tp53inp1 LOF group, versus controls. (**G**) Quantification revealed no significant difference in the proportions of cells displaying immunoreactivities corresponding to proliferating cells (Ki67/EdU) and NPCs (Nestin/Sox2) in Tp53inp1 LOF group, compared to controls. (**H**) A schematic representation of Cdkn1a LOF approach; single NPCs were transduced with either Cdkn1a siRNA or scrambled control and subjected to proliferation for 3 DIV. (**I**) The transfection efficiency of Cdkn1a siRNA was tested by qPCR. (**J**) The size of neurospheres increased in the Cdkn1a LOF group, compared to controls. (**K**) qPCR analysis of transduced cells revealed a significant increase in levels of *Ki67*/*Nestin*/*Sox2* transcripts in Cdkn1a LOF group versus controls. (**L**) Immunofluorescence analysis of transduced cells after 3 DIV revealed increase in proliferating NPCs-specific immunoreactivities in Cdkn1a LOF group, versus controls. (**M**) Immunofluorescence analysis of transduced cells after 3 DIV revealed increase in EdU immunoreactivities in Cdkn1a LOF group, versus controls. (**N**) Quantification revealed significant increase in the proportions of cells displaying immunoreactivities corresponding to proliferating cells (Ki67/EdU) and NPCs (Nestin) in Cdkn1a LOF group, compared to controls. Scale bar, 400 μm (C, J) and 50 μm (E, F, L, M). Amplification curves and gene expressions were normalized to the house-keeping gene GAPDH (for mRNA) and U6 snRNA (for miRNA). Data are mean ± sd. Experiments were carried out three times in triplicates for *in vitro* perturbation.

**
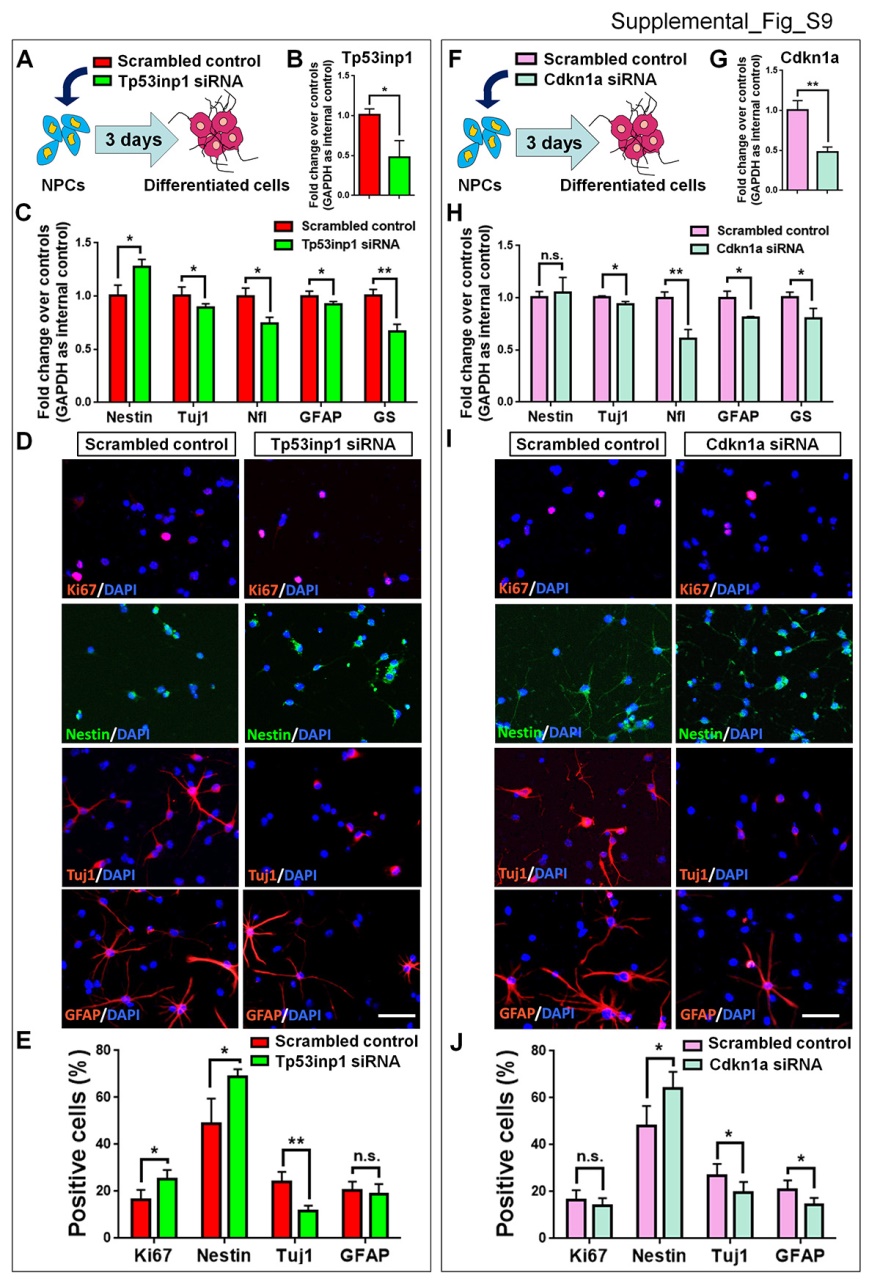
**

**Figure S9. Tp53inp1 and Cdkn1a siRNAs inhibit the differentiation of NPCs.**

(**A**) A schematic representation of Tp53inp1 LOF approach; single NPCs were transduced with either Tp53inp1 siRNA or scrambled control and subjected to differentiation for 3 DIV. (**B**) The transfection efficiency of Tp53inp1 siRNA was tested by qPCR. (**C**) qPCR analysis of transduced cells revealed a decrease and an increase in the levels of transcripts corresponding to markers of NPCs (*Nestin*) and differentiated cells (*Tuj1*/*Nfl*/*GFAP*/*GS*), respectively, in Tp53inp1 LOF group versus controls. (**D**) Representative immunofluorescence with anti-Ki67, anti-Nestin, anti-Tuj1 and anti-GFAP for *miR-106a* inhibitor- or scrambled inhibitor control-transduced cells after 3 DIV. (**E**) Quantification revealed a significant decrease and increase in the proportions of cells displaying Ki67/Nestin and Tuj1/GFAP immunoreactivities, respectively, in *miR-106b* LOF group, compared to controls. (**F**) A schematic representation of *miR-106b* GOF approach; single NPCs were transduced with either *miR-106b* mimics or scrambled mimics control and subjected to differentiation for 3 DIV. (**G**) The transfection efficiency of Cdkn1a siRNA was tested by qPCR. (**H**) qPCR analysis of transduced cells revealed a significant decrease in the levels of transcripts corresponding to markers of differentiated cells (*Tuj1*/*Nfl*/*GFAP*/*GS*), in Cdkn1a LOF group versus controls. (**I**) Representative immunofluorescence with anti-Ki67, anti-Nestin, anti-Tuj1 and anti-GFAP for Cdkn1a siRNA- or scrambled control-transduced cells after 3 DIV. (**J**) Quantification revealed a significant decrease and increase in the proportions of cells displaying Ki67/Nestin and Tuj1/GFAP immunoreactivities, respectively, in Cdkn1a LOF group, compared to controls. Scale bar, 50 μm (D, I). Amplification curves and gene expressions were normalized to the house-keeping gene GAPDH (for mRNA) and U6 snRNA (for miRNA). Data are mean ± sd. Experiments were carried out three times in triplicates for *in vitro* perturbation.

**
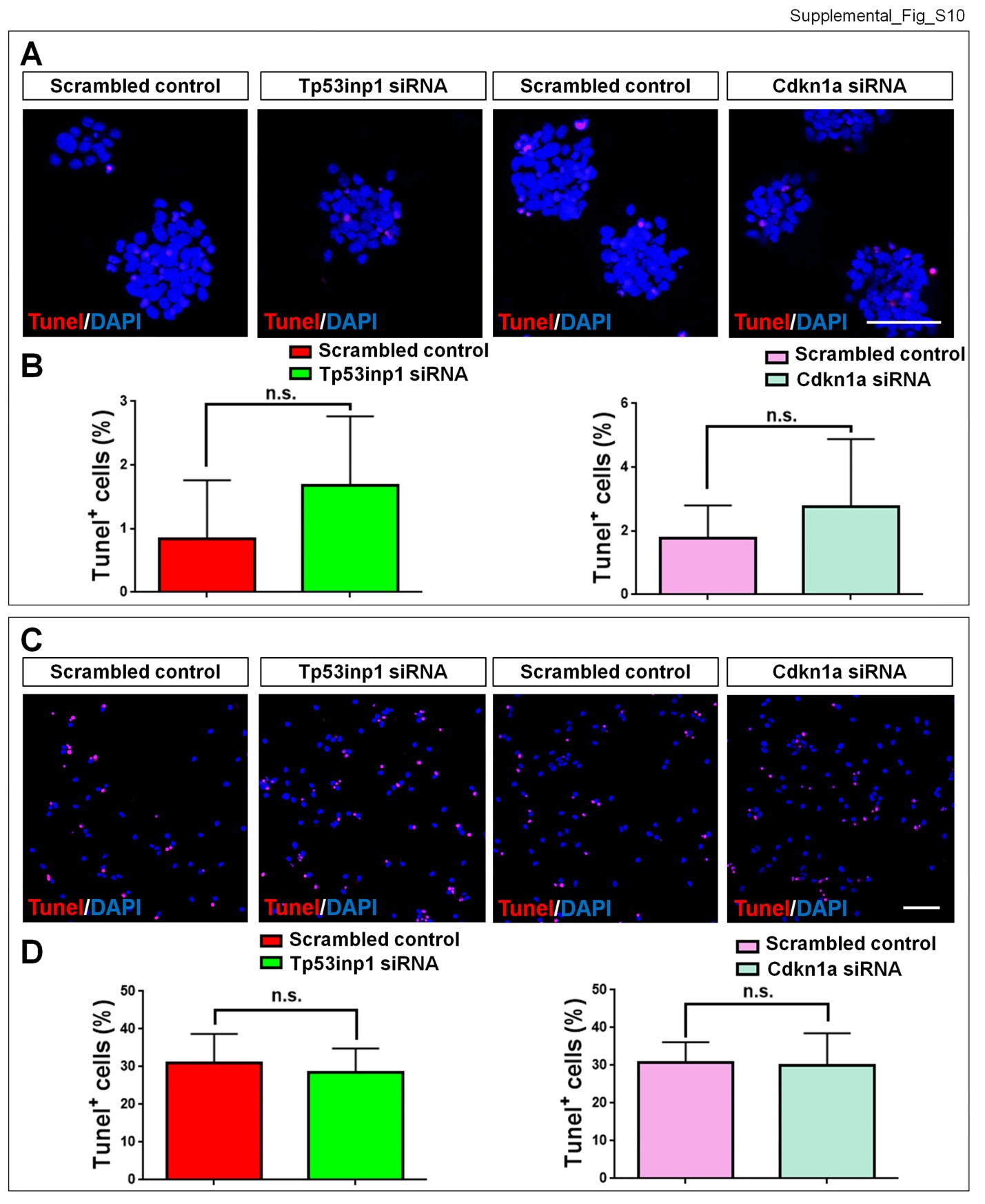
**

**Figure S10. Tp53inp1 and Cdkn1a have no effect on NPCs survival.**

(**A**) Representative pictures of Tunel+ (red) cells in scrambled control-, Tp53inp1 siRNA- and Cdkn1a siRNA-transfected NPCs after 3DIV in proliferation conditions were shown. (**B**) Quantification revealed no significant difference in the proportions of Tunel+ cells in both Tp53inp1 and Cdkn1a siRNA treated groups, compared to scrambled control groups. (**C**) Representative pictures of Tunel+ (red) cells in scrambled control-, Tp53inp1 siRNA- and Cdkn1a siRNA-transfected NPCs after 3DIV in differentiation conditions were shown. (**D**) Quantification revealed no significant difference in the proportions of Tunel+ cells in both Tp53inp1 and Cdkn1a siRNA treated groups, compared to scrambled control groups. Scale bar, 50 μm (A, C). Data are mean ± sd. Experiments were carried out three times in triplicates for *in vitro* perturbation.

**Table S1. List of specific primers.**

| Gene | Sequence | Size (bp) | T^o^ | Accession N. |
| --- | --- | --- | --- | --- |
| *β-tubulin* | 5’-CTTTATCTTCGGTCAGAGTGGTGC-3’  5’-TTCTTTCCGCACGACATCTAGG-3’ | 103 | 57 | NM_023279.2 |
| *Bim* | 5’-GGATTCACACCACCTCCGCT-3’  5’-GTCGGGATTACCTTGCGGTT-3’ | 254 | 57 | NM_207680.2 |
| *Cdkn1a* | 5’-GAATGGAGACAGAGACCCCAG-3’  5’-GACAACGGCACACTTTGCTC-3’ | 131 | 56 | NM_001111099.2 |
| *GAPDH* | 5’-CATGTTCCAGTATGACTCCACTC-3’  5’-GGCCTCACCCCATTTGATGT-3’ | 136 | 60 | NM_001289726.1 |
| *GFAP* | 5’-TTGCTGGAGGGCGAAGAAAA-3’  5’-CATCCCGCATCTCCACAGTC-3’ | 148 | 57 | NM_010277.3 |
| *GS* | 5’-TCACAGGGACAAATGCCGAG-3’  5’-GTTGATGTTGGAGGTTTCGTGG-3’ | 362 | 58 | NM_008131 |
| *Ki67* | 5’-CAGCAGAAGAATCGTGGGAGAC-3’  5’-CCTACTTTGGGTGAAGAGGTTGC-3’ | 103 | 54 | NM_001081117.2 |
| *Lhx6* | 5’-GACAGCTCCGAGCCAGTTC-3’  5’-ACATGGGGAGGCTCTACCTT-3’ | 196 | 56 | NM_010713.2 |
| *NeuroD2* | 5’-TCCTCCCTTCTCTCGTGGAG-3’  5’-CCCTTACCAACTGCCTCTGG-3’ | 129 | 57 | NM_010895.3 |
| *Nestin* | 5’-GGAGAGTCGCTTAGAGGTGC-3’  5’-TCAGGAAAGCCAAGAGAAGC-3’ | 327 | 57 | NM_016701.3 |
| *Nfl* | 5’-GCAGGACACAATCAACAAACTGG-3’  5’-GCTTTCGTAGCCTCAATGGTCTC-3’ | 348 | 56 | NM_031783 |
| *Ngn2* | 5’-AGAAGACCCGCAGGCTCAAG-3’  5’-CGTGGAGTTGGAGGATGACG-3’ | 341 | 57 | NM_009718.2 |
| *Ngn3* | 5’-GAGCGCAATCGCATGCACAA-3’  5’-GCTCCGGGCCATAGAAGCTG-3’ | 178 | 57 | NM_009719.6 |
| *Nr4A3* | 5’-TGACTCTCCCCCAATCCAGA-3’  5’-GCAGGGCATATCTGGAGGGTA-3’ | 264 | 57 | NM_015743.3 |
| *p53* | 5’-CATGAGCGCTGCTCCGATGG-3’  5’-AATTTCCTTCCACCCGGATAA-3’ | 67 | 57 | NM_011640.3 |
| *Pten* | 5’-TTAGCCTCTTGATGTGTGCAT-3’  5’-TGGTAGCCAAACGGAACTTCAT-3’ | 103 | 57 | NM_008960.2 |
| *Runx1* | 5’-TGGCACTCTGGTCACCGTCAT-3’  5’-GAAGCTCTTGCCTCTACCGC-3’ | 142 | 57 | NM_009821.3 |
| *Runx3* | 5’-TTCATCCAGGCTGCTGAGTCT-3’  5’-TCCCAGTCCCACAGGAATGT-3’ | 146 | 57 | NM_019732.2 |
| *Smad7* | 5’- GAATCTTACGGGAAGATCAAC-3’  5’- CACAGAGTCGACTAAGGT-3’ | 67 | 57 | NM_001042660.1 |
| *Sox2* | 5’-CCCAGCGCCCGCATGTATAA-3’  5’-GCGCTTGCTGATCTCCGAGT-3’ | 240 | 57 | NM_011443.4 |
| *Timp2* | 5’-CAGGTACCAGATGGGCTGTG-3’  5’-TGGTGCCCATTGATGCTCTT-3’ | 123 | 57 | NM_011594.3 |
| *Tp53inp1* | 5’-AAGTGGTCCCAGAATGGAAGC-3’  5’-GCTGGAAGGAGACAGCACTT-3’ | 353 | 55 | NM_021897.3 |
| miRNA |  |  |  |  |
| Universal primer | 5’-GAATCGAGCACCAGTTACGC-3’ |  |  |  |
| *U6* | 5’-TGGCCCCTGCGCAAGGATG-3’ |  | 55 |  |
| *let-7b* | 5’-TGAGGTAGTAGGTTGTGTGGTT-3’ |  | 55 | MIMAT0000522 |
| *let-7c* | 5’-TGAGGTAGTAGGTTGTATGGTT-3’ |  | 55 | MIMAT0000776 |
| *miR-101a* | *5’-*TACAGTACTGTGATAACTGAA*-3’* |  | 55 | MIMAT0000133 |
| *miR-106a* | *5’-*CAAAGTGCTAACAGTGCAGGTAG*-3’* |  | 55 | MIMAT0000385 |
| *miR-106b* | *5’-*TAAAGTGCTGACAGTGCAGAT*-3’* |  | 55 | MIMAT0000386 |
| *miR-124a* | *5’-*TAAGGCACGCGGTGAATGCC*-3’* |  | 55 | MIMAT0000716 |
| *miR-125a* | *5’-* TCCCTGAGACCCTTTAACCTGTGA*-3’* |  | 55 | MIMAT0000135 |
| *miR-130a* | *5’-*CAGTGCAATGTTAAAAGGGCAT*-3’* |  | 55 | MIMAT0000141 |
| *miR-141* | *5’-*TAACACTGTCTGGTAAAGATGG*-3’* |  | 55 | MIMAT0000153 |
| *miR-145a* | *5’-*GTCCAGTTTTCCCAGGAATCCCT*-3’* |  | 55 | MIMAT0000157 |
| *miR-146a* | *5’-*TGAGAACTGAATTCCATGGGTT*-3’* |  | 55 | MIMAT0000158 |
| *miR-153* | *5’-*TTGCATAGTCACAAAAGTGATC*-3’* |  | 55 | MIMAT0000163 |
| *miR-155* | *5’-*TTAATGCTAATTGTGATAGGGGT*-3’* |  | 55 | MIMAT0000165 |
| *miR-17* | *5’-*CAAAGTGCTTACAGTGCAGGTAG*-3’* |  | 55 | MIMAT0000386 |
| *miR-18a* | *5’-*TAAGGTGCATCTAGTGCAGATAG*-3’* |  | 55 | MIMAT0000528 |
| *miR-181c* | *5’-*AACATTCAACCTGTCGGTGAGT*-3’* |  | 55 | MIMAT0000674 |
| *miR-182* | *5’-*TTTGGCAATGGTAGAACTCACACCG*-3’* |  | 55 | MIMAT0000211 |
| *miR-185* | *5’-*TGGAGAGAAAGGCAGTTCCTGA*-3’* |  | 55 | MIMAT0000214 |
| *miR-19a* | *5’-*TGTGCAAATCTATGCAAAACTGA*-3’* |  | 55 | MIMAT0000651 |
| *miR-193a* | *5’-*AACTGGCCTACAAAGTCCCAGT*-3’* |  | 55 | MIMAT0000223 |
| *miR-193b* | *5’-*AACTGGCCCACAAAGTCCCGCT*-3’* |  | 55 | MIMAT0004859 |
| *miR-199a* | *5’-*CCCAGTGTTCAGACTACCTGTTC*-3’* |  | 55 | MIMAT0000229 |
| *miR-20a* | *5’-*TAAAGTGCTTATAGTGCAGGTAG*-3’* |  | 55 | MIMAT0000529 |
| *miR-200b* | *5’-*TAATACTGCCTGGTAATGATGA*-3’* |  | 55 | MIMAT0000233 |
| *miR-203b* | *5’-*GTGAAATGTTTAGGACCACTAG*-3’* |  | 55 | MIMAT0000236 |
| *miR-204* | *5’-*TTCCCTTTGTCATCCTATGCCT*-3’* |  | 55 | MIMAT0000237 |
| *miR-21a* | *5’-*TAGCTTATCAGACTGATGTTGA*-3’* |  | 55 | MIMAT0000530 |
| *miR-21b* | *5’-*TAGTTTATCAGACTGATATTTCC*-3’* |  | 55 | MIMAT0025121 |
| *miR-21c* | *5’-*TAGCTTATCAGACTGGTACAA*-3’* |  | 55 | MIMAT0025148 |
| *miR-216a* | *5’-*TAATCTCAGCTGGCAACTGTGA*-3’* |  | 55 | MIMAT0000662 |
| *miR-22* | *5’-*AAGCTGCCAGTTGAAGAACTGT*-3’* |  | 55 | MIMAT0000531 |
| *miR-23b* | *5’-*ATCACATTGCCAGGGATTACC*-3’* |  | 55 | MIMAT0000125 |
| *miR-25* | *5’-*CATTGCACTTGTCTCGGTCTGA*-3’* |  | 55 | MIMAT0000652 |
| *miR-26b* | *5’-*TTCAAGTAATCCAGGATAGGCT*-3’* |  | 55 | MIMAT0000533 |
| *miR-27b* | *5’-*TTCACAGTGGCTAAGTTCCGC*-3’* |  | 55 | MIMAT0000537 |
| *miR-29b* | *5’-*TAGCACCATTTGAAATCAGTGTT*-3’* |  | 55 | MIMAT0000127 |
| *miR-30a* | *5’-*TGTAAACATCCTCGACTGGAAG*-3’* |  | 55 | MIMAT0000128 |
| *miR-323* | *5’-*CACATTACACGGTCGACCTCT*-3’* |  | 55 | MIMAT0000551 |
| *miR-34a* | *5’-*TGGCAGTGTCTTAGCTGGTTGT*-3’* |  | 55 | MIMAT0000542 |
| *miR-340-5p* | *5’-*TTATAAAGCAATGAGACTGATT*-3’* |  | 55 | MIMAT0004651 |
| *miR-363* | *5’-*AATTGCACGGTATCCATCTGTA*-3’* |  | *55* | MIMAT0000708 |
| *miR-672* | *5’-*TGAGGTTGGTGTACTGTGTGTGA*-3’* |  | 55 | MIMAT0003735 |
| *miR-9* | *5’-*TCTTTGGTTATCTAGCTGTATGA*-3’* |  | 55 | MIMAT0000142 |
| *miR-92a* | *5’-*TATTGCACTTGTCCCGGCCTG*-3’* |  | 55 | MIMAT0000539 |
| *miR-93* | *5’-*CAAAGTGCTGTTCGTGCAGGTAG*-3’* |  | 55 | MIMAT0000540 |

**Table S2. List of primary antibodies**


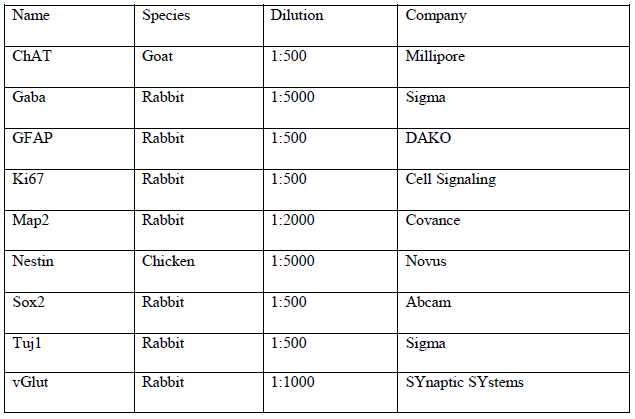

Supplement: Supplementary file 1 — Figure S1. miR-106a does not promote the proliferation of NPCs. Figure S2. miR-106a does not regulate the differentiation of NPCs. Figure S3. miR-106b has no effect on neuronal subtype specification. Figure S4. miR-106b has no effect on NPCs’ survival. Figure S5. miR-106a has no effect on NPCs’ survival. Figure S6. miR-106b regulates the differentiation of NPCs in vivo. Figure S7. The validation of Tp53inp1 and Cdkn1a siRNAs. Figure S8. Tp53inp1 and Cdkn1a siRNAs promote the proliferation of NPCs. Figure S9. Tp53inp1 and Cdkn1a siRNAs inhibit the differentiation of NPCs. Figure S10. Tp53inp1 and Cdkn1a have no effect on NPCs’ survival. Table S1. List of specific primers. Table S2. List of primary antibodies. (DOCX 4511 kb) [file 13287_2019_1387_MOESM1_ESM.docx]
